# Global Estimates of the HPV-related, Noncervical Cancer Burden in People With HIV and AIDS and the Potential Effect of Improved HPV Vaccine Coverage in This Population: A Systematic Review, Meta-analysis and Modeling Study

**DOI:** 10.1093/ofid/ofaf577

**Published:** 2025-09-16

**Authors:** Namwa Wongkalasin, Victoria Pilkington, Jacob Levi, Toby Pepperrell, Anna Garratt, Cassandra Fairhead, Andrew Hill

**Affiliations:** School of Public Health, Imperial College London, London, UK; School of Public Health, Imperial College London, London, UK; School of Public Health, Imperial College London, London, UK; School of Public Health, Imperial College London, London, UK; Biological and Environmental Sciences Department, University of Stirling, UK; Department of Medicine, Cardiff and Vale University Health Board, Cardiff, UK; Acute Medicine, Royal Free Hospital, London, UK; Department of Pharmacology and Therapeutics, University of Liverpool, Liverpool, UK

**Keywords:** epidemiology, HIV, human papilloma virus, non-cervical, vaccines

## Abstract

**Background:**

While HIV/HPV coinfection significantly increases cervical carcinogenesis, the relationship with HPV-related noncervical cancers is less understood. HPV vaccination is key in prevention, but global coverage is low (21%). We aimed to quantify the risk of noncervical cancers among people living with HIV and AIDs (PWHA), estimate this cancer burden globally, and the impact of improved HPV vaccine coverage.

**Methods:**

We conducted a systematic review and meta-analysis of 3 databases to identify studies comparing HPV-related noncervical cancer risk in PWHA and HIV negative populations, generating a risk ratio for each cancer. This was combined with country-specific HIV prevalence and the incidence of cancer cases to calculate the burden. Vaccine impact was estimated using vaccine coverage data and point efficacy estimates.

**Results:**

25 studies were included analyzing 1 560 923 million PWHA. The risk of developing each HPV-related noncervical cancer was significantly increased in PWHA. The pooled risk was 30.32 (CI 20.54–44.75) for anal, 15.78 (CI 4.62–53.92) for vaginal, 6.99 (CI 3.37–14.51) for vulvar, 6.37 (CI 3.98–10.21) for penile, and 2.74 (CI 2.10–3.58) for oropharyngeal cancers. Globally, 30% of cases in Africa were HIV-attributable, compared with 5% worldwide. If HPV vaccination coverage increased to 90%, we could prevent 1987 more cancer cases, assuming 50% vaccine efficacy.

**Conclusions:**

This meta-analysis confirms that PWHA are more susceptible to adverse outcomes beyond cervical cancer and must be prioritized in HPV prevention and vaccine efficacy research. In countries with high HIV/HPV coinfection prevalence, integrating HPV vaccination with HIV services could minimize disparities in the cancer burden worldwide.

## Teaser

This research highlights people living with HIV and AIDS (PWHA) as a high-risk group for noncervical HPV-related cancers, supporting prioritization of this population in vaccination. Improving vaccine coverage in PWHA could minimize the cancer burden in high HIV prevalence countries.

In 2022, there were over 920 000 cancer cases related to human papillomavirus (HPV) worldwide [1]. Of these, 77% occurred in low- and middle-income countries (LMICs) where 80% of the world's population reside, revealing a disparity in the global cancer distribution [1]. As the incidence of HPV-related, noncervical cancers have risen by around 22% since 2018, renewed efforts are needed to address this gap and direct resources appropriately [2].

Although most HPV infections are transient, persistent viruses can result in long-term sequelae [2]. Evidence shows that almost all cervical cancer cases (99.7%) are caused by high-risk HPV infection [3]. In 2020, the World Health Organization (WHO) announced the “Global Strategy to accelerate elimination of cervical cancer,” placing HPV high on the research agenda [3]. However, the role of HPV in noncervical malignancies is hugely overlooked in comparison [2, 4]. HPV is implicated in anal (90%), oropharyngeal (70%), vaginal (75%), vulvar (69%), and penile (63%) cancers [5, 6].

While these cancers are less common than cervical, their incidence and mortality are rising, causing over 114 000 deaths in 2022 [1]. Oncogenesis following HPV infection is exacerbated by other exposures including HIV [2]. HIV-induced immunosuppression reduces an individual's ability to combat the HPV virus, increasing the likelihood of cancer progression [7]. One analysis estimated that 63% of people with cervical cancer in Southern Africa were living with HIV [8]. This high coinfection rate may extend to noncervical sites of HPV infection, but there is a paucity of research in this area. Further, evidence indicates a synergistic relationship between the viruses; meaning that public health measures tackling one infection could bring benefits for the other [9].

The WHO recommends 3 measures: vaccinate 90% of girls under 15, screen 70% of women for cervical cancer by age 45, and treat 90% of precancerous lesions [3]. However, screening programs for noncervical, HPV-related cancers have not yet been established and for many LMICs, implementing screening and treatment may not be feasible due to low resource health systems [10]. Since noncervical subtypes comprised a third (264 478) of the HPV-related cancer burden (925 522) in 2022, vaccines designed primarily for cervical cancer should be utilized beyond this intention [1, 11].

Currently, the WHO prioritizes vaccinating people living with HIV and AIDS (PWHA) in immunization programs due to the known increased risk of HPV acquisition and cancer development [3]. Although, in areas that lack sufficient vaccine supplies, PWHA are often overlooked [8]. Regarding the epidemiological burden, literature has estimated this for cervical and anal cancer, although the latter used data from the United States limiting transferability to LMIC contexts [8, 12]. A more comprehensive view of the global number of noncervical HPV cancers related to HIV and AIDS could guide the mobilization of resources and raise awareness for this under researched population. Assessing the effect of vaccination on this burden may inform prevention initiatives in LMICs which experience high rates of HIV; this could minimize the gross inequity in the worldwide HPV cancer burden.

Thus, we aimed to quantify the global number of HPV-related, noncervical cancers attributable to HIV and AIDS, and estimate the effect of upscaling vaccination access through three objectives:

1. Performing a systematic review and meta-analysis to generate a pooled risk for developing noncervical, HPV-related cancers among PWHA compared with HIV negative populations.
2. Combining this risk with the Joint United Nations Program on HIV and AIDS (UNAIDS) HIV prevalence and the International Agency for Research on cancer's (IARC) 2022 dataset to calculate the estimated number of noncervical, HPV-related cancer cases among PWHA worldwide [1, 13].
3. Applying vaccine efficacy and WHO coverage estimates to model the impact of improved vaccination on preventing noncervical, HPV cancers in PWHA [14].

## METHODS

This review follows the Preferred Reporting Items for Systematic Reviews and Meta-analyses for Protocols (PRISMA) guidelines [15].

We conducted a systematic search of MEDLINE, EMBASE, and Global Health databases to identify studies observing the association between HIV or AIDS and HPV-related, noncervical cancers. A combination of Medical Subject Headings or key words related to “HIV,” HPV,” each “noncervical cancer,” and epidemiological search terms were used including “incidence,” “prevalence,” and “incidence rate.” No date or language restrictions were applied. Additional hand searches of gray literature including references of suitable articles were further screened to improve the comprehensiveness of the search. The full details of the search strategy can be found in the Supplementary Appendix Table 1.

We included published, observational studies reporting the risk of developing HPV-related, noncervical cancers in PWHA, compared with HIV negative controls or the general reference population. We considered the following noncervical cancers to be HPV-related based on the existing literature: anal, oropharyngeal, penile, vulvar, and vaginal cancers [5]. Papers were stratified according to the site-specific cancer outcome they reported. The predefined selection criteria are outlined in Supplementary Appendix Table 2.

All identified papers were uploaded onto COVIDENCE and duplicates removed. The remaining abstracts were screened against the predefined inclusion criteria, followed by full text screening in duplicate. We extracted key data on the observed number of incident cases for each specific cancer site, the total number of PWHA, and the overall effect measure. Study-level characteristics including study type, setting, publication year, country, and sample size were recorded along with population demographics. For studies reporting incidence from the same registry or cohort, only 1 was included to avoid overlapping populations and double inclusion; those with the longest follow up time or largest cohort were chosen. For registry studies reporting segregated time periods, we included data for only the population or period studied.

We used the Newcastle–Ottawa scale to assess the methodological quality of included studies [16]. Studies were considered medium risk of bias if they scored <7 stars, and high risk if rated 5 stars or lower.

### Data Sources

For the modeling, 3 data sources were used. HIV prevalence for adults aged ≥ 15 years in 2022 was collected from UNAIDS [13]. Cancer incidence in 2022 was obtained from the IARC database [1, 11]. Country-specific, regional, and global estimates per 100 000 people were extracted. Cancers included oropharyngeal, anal, penile, vaginal and vulvar as defined by the International Classification of Diseases 10th revision codes [17]. On combining the data sources, estimates for 169 countries were available. Country-specific estimates for vaccine coverage in 2022 were collected from WHO's database [14].

### Statistical Analysis

An inverse variance meta-analysis was conducted to generate a pooled risk estimate for PWHA compared with HIV negative populations for each cancer. We regarded risk ratio (RR), incidence rate ratio, and standardized incidence ratio (SIR) estimates as equivalent measures, given that each cancer is considered rare within the general population [18]. The meta-analysis was carried out with a random effects model, due to the high potential for heterogeneity. Interstudy heterogeneity was quantified through an *I*^2^ parameter and considered substantial if over 50%, according to Cochrane guidelines [19]. Subgroup analyses of study types were conducted to explore sources of heterogeneity. Further sensitivity analyses and funnel plots were conducted to assess publication bias.

We combined our pooled risk estimate for PWHA with country specific UNAIDS HIV prevalence, to calculate the population-attributable fraction (PAF, %) to HIV for each country using Levin's formula [18]. This estimates the fraction of cases in a population that is “attributable” to an exposure (HIV), through a causal interpretation of the number of cases that would not have occurred if no exposure was present [18]. We then multiplied the number of incident cases from the IARC database by the PAF to generate the incidence for each cancer attributable to HIV and AIDS in 2022 [1]. This analysis was conducted at the country-specific level and combined into regional and global estimates.

The modeling combined country-specific vaccine coverage data with vaccine efficacy estimates to generate the number of cancer cases in PWHA that could be averted by increasing vaccine coverage (Supplementary Appendix Figure 1) [14]. Based on the inconclusive data on vaccine efficacy in PWHA, estimates of 50%, 70%, and 90% were chosen to represent the potential spectrum [20, 21]. A more in-depth description of methods and modeling assumptions are in the Supplementary Appendix.

All statistical analysis was performed using R software, specifically the “meta” package (version 4.3.1).

## RESULTS

**Figure 1.**
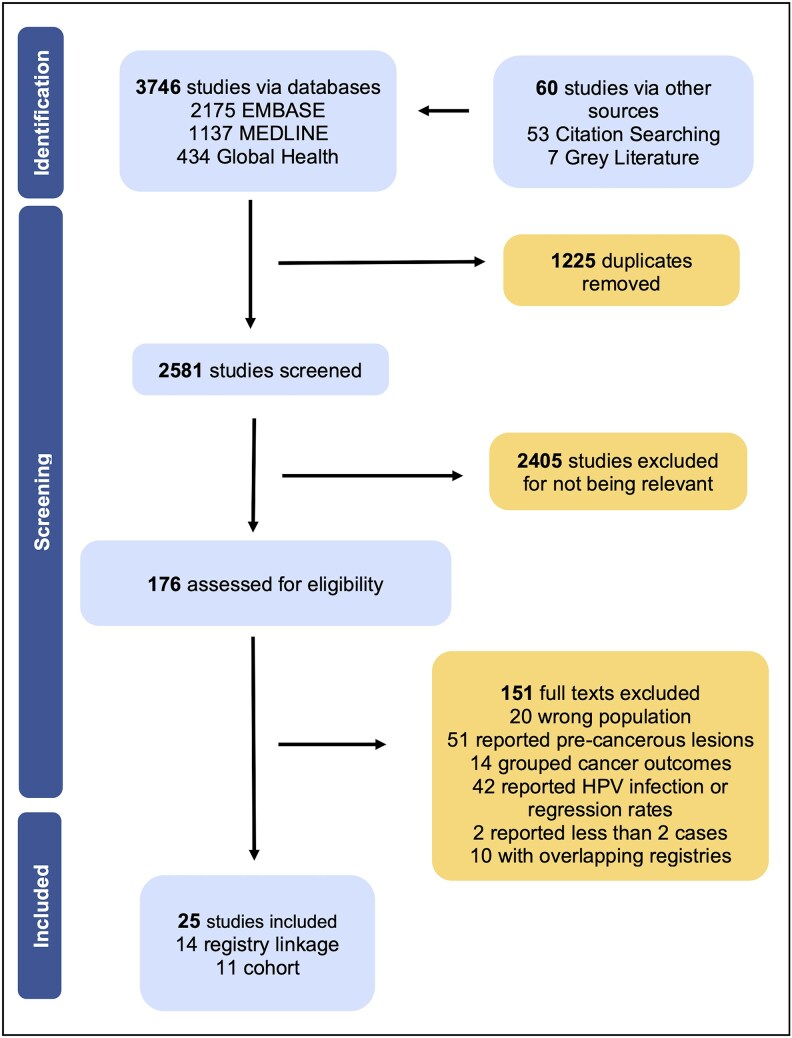
PRISMA diagram depicting the study selection process.

Twenty-five publications were included following the screening process outlined in Figure 1 (Supplementary references in Appendix). Altogether, these papers studied 1 560 923 PWHA and reported 3227 cases of HPV-related noncervical cancer, with study periods ranging from 1978 to 2017. A description of each study's characteristics is in Supplementary Appendix Table 3, and summary of the results can be found in Supplementary Appendix Table 4. Most studies (94%) were conducted in HICs, mainly North America and Europe, except 2 studies from Brazil (*n* = 1) and China (*n* = 1). The mean antiretroviral therapy (ART) coverage was 58.01% and mean age of entry was 40.67 years. Around 56% of the studies were registry linkage (*n* = 14), followed by cohort studies (*n* = 11). Six studies were deemed moderate risk of bias and 5 high, with a median score of 6.12 out of 9 stars on the Newcastle Ottawa scale. A full assessment of methodological quality is in Supplementary Appendix Table 5.

### Risk of Noncervical Cancers in People With HIV and AIDS

Overall, 48 risk estimates comparing PWHA to HIV negative populations were extracted from the 35 studies. This included 17 studies for anal, 14 for oropharyngeal, 4 for vulvar, 4 for vaginal, and 11 for penile cancers respectively.

**Figure 2.**
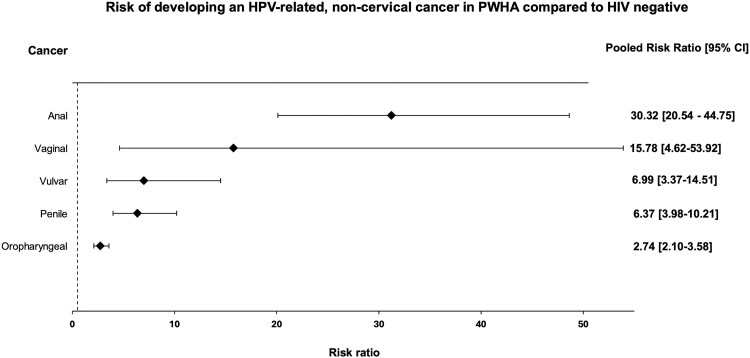
Summary forest plot displaying overall summary risk estimates for each HPV-related noncervical cancer. Risk rations are comparing the risk of developing cancer in PWHA compared with the HIV negative populations.

Meta-analysis of risk estimates generated an overall pooled RR of developing each HPV-related noncervical cancer in PWHA compared with HIV negative populations (Figure 2). Full forests plots for each cancer are found in Supplementary Appendix Figure 2.

The highest RR was seen in anal cancer (RR 30.32, 95% CI 20.54–44.75) whereas the lowest was oropharyngeal (RR 2.74, 95% CI 2.10–3.58). The remaining anogenital cancers had elevated rates, although fewer studies reported these outcomes: vulvar (RR 6.99, 95% CI 3.37–14.51), vaginal (RR 15.78, 95% CI 4.62–53.92), and penile (RR 6.37, 95% CI 3.98–10.21). Overall, all 5 cancers occurred at increased rates compared with HIV negative populations, though the risk varied depending on cancer subtype.

Interstudy heterogeneity was high across all analyses ranging from *I*^2^ = 91.6% (*P* < .0001) for anal cancer to *I*^2^ = 73.8% (*P* < .0001) for oropharyngeal cancer. Subgroup analysis by study design, for example registry linkage, demonstrated a reduction in heterogeneity, and following a χ^2^ analysis subgroup differences were not statistically significant. Two sensitivity analyses were conducted by removing studies deemed high risk of bias and published before 2014, respectively, which demonstrated no significant changes (Supplementary Appendix Table 6). The funnel plots for anal and oropharyngeal cancers demonstrated asymmetry with bias toward studies reporting greater positive associations between HIV and HPV-related cancer (Supplementary Appendix Figure 3).

### Global Burden of Noncervical, HPV-related Cancers Attributable to HIV and AIDS

**Figure 3.**
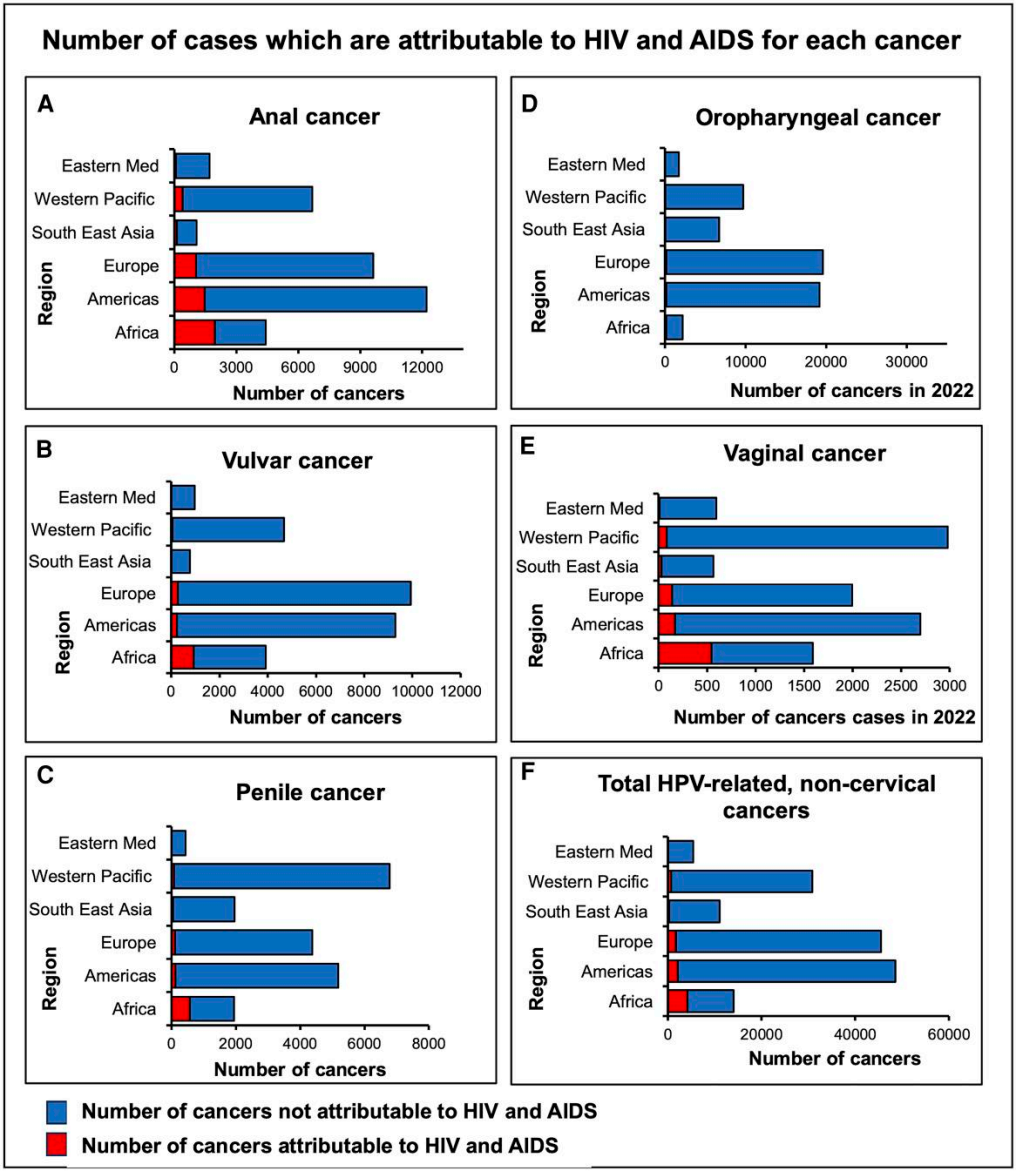
Number of cases which are attributable to HIV and AIDS for each cancer and region worldwide. (*A–E*) Shows the number of specific cancers (Anal, Oropharyngeal, Penile, Vulvar, and Vaginal) attributable to HIV worldwide. (*F*) Shows total number of HPV-related, noncervical cancers attributable to HIV worldwide.

**Figure 4.**
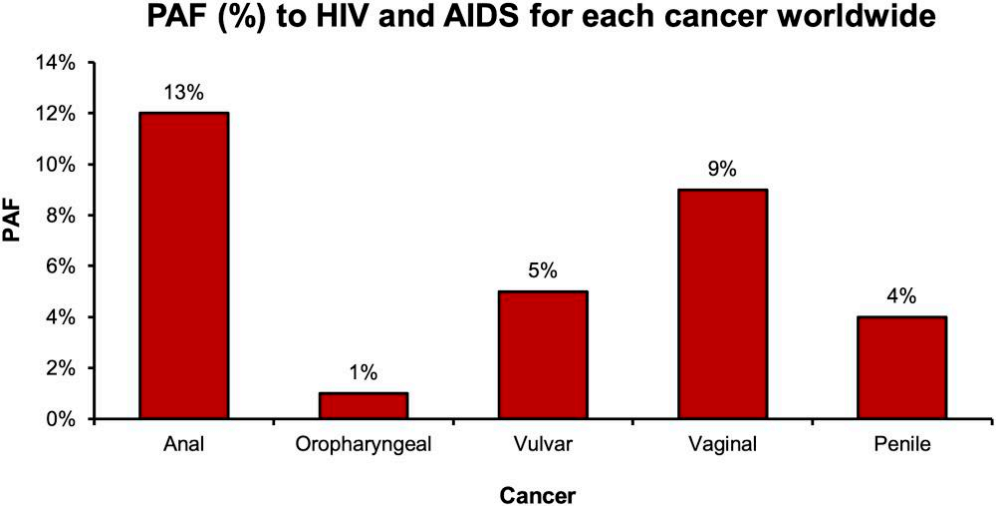
Population attributable fraction to HIV and AIDS for each cancer.

In summary, the number of noncervical, HPV-related cancers attributable to HIV and AIDS exhibited differences depending on cancer and geographical region (Figure 3). For anal cancer, an estimated 35 719 cases occurred worldwide in 2022; around 13.8% of these were attributable to HIV (Figure 4). This HIV-attributable fraction ranged from 3.7% in the Eastern Mediterranean, to 12% in the Americas, to more than 44.2% in the African region. These geographical variations were echoed for all cancers subtypes in absolute terms.

Of the 50 countries with the highest-ranking PAFs to HIV, 37 were in Africa (Supplementary Appendix Figure 4). The remainder were divided between the Americas (*n* = 10), the Western Pacific region (*n* = 1), Southeast Asia (*n* = 1), and Europe (*n* = 1). The 5 countries with the highest PAFs, in order, were Eswatini, Lesotho, Botswana, South Africa, and Namibia. For anal cancer, between 78.7% and 88.9% of diagnosed cases within these countries were HIV and AIDS attributable. The remaining cancers exhibited the same geographical distribution, although the PAFs were lower for these cancers. In Eswatini, for example, 80.8% of vaginal, 62.1% of vulvar, 59.3% of penile, and 32.1% of oropharyngeal cancers diagnosed in 2022 were attributable to HIV.

### Vaccination Model Results

**Figure 5.**
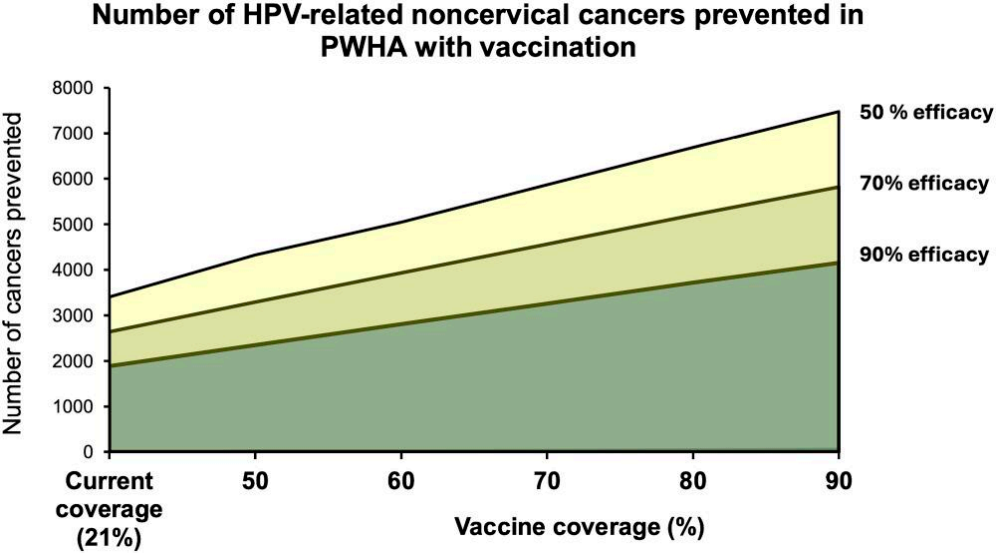
Approximate modeling of the number of cancers, which may be prevented in people presently living with HIV by improving vaccination coverage up to 90% level. Three scenarios were estimated using point vaccine efficacy estimates of 50%, 70%, and 90%.

Our model shows that 3384, noncervical, HPV-related cancers per year may be prevented among PWHA at the current vaccine coverage rates (Figure 5). This estimate assumes that vaccine protection extends to men via herd immunity mechanisms.

If vaccine coverage increases to the WHO's 90% target, we predict that more than twice the number of cases could be averted (7462). This is estimated with the 90% efficacy scenario. As efficacy reduces, the number of averted cancers falls incrementally, but even with a 50% efficacy scenario, 1987 more cancer cases will be prevented if coverage is increased to 90%.

## DISCUSSION

This systematic review and meta-analysis of over 1.5 million PWHA demonstrates that PWHA face a significantly higher risk of developing all HPV-related, noncervical cancers compared with their HIV-negative peers. Our analysis expands our understanding of the risk of such cancers across the entire spectrum of HIV infection, which is important due to the increased longevity of PWHA owing to accessible ART.

The anogenital subtypes exhibited the highest estimates, particularly anal cancer with a 30-fold increased risk. For the remaining cancers, the risk is as high as around 15 times for vaginal, 7 for vulvar, 6 for penile, and 2 times for oropharyngeal. In 2022, we estimate around 14% of incident anal cancers were attributable to HIV and AIDS infection worldwide. Given the disparities in HIV prevalence, significant geographical variations were seen for all analyzed cancers, with most cases concentrated in Africa. Our model projected that globally 7462 noncervical cancers per year could be averted in PWHA if vaccine coverage improves to 90%.

In our epidemiological analysis, of the 50 countries with the highest PAFs, only 15 were located outside Africa (Supplementary Appendix Figure 3). This variation stems from factors including HIV endemicity and healthcare provision, including vaccination [2, 12]. We found that 70% of the top 50 countries were LMICs. Given the practicalities of establishing cancer screening and treatment, vaccines remain the most affordable yet impactful choice [10]. Abbas et al found that vaccinating 1000 people could avert 2 cervical cancer deaths in Switzerland compared with 34 in Tanzania, highlighting the need for contextualized approaches to HPV prevention [22]. Thus, international agencies alongside countries with high HPV/HIV coinfection rates should consider this concurrent burden when planning vaccination programs.

Key factors, which may have contributed to the wide variations in PAFs to HIV seen in Supplementary appendix Figure 4, include varying capacities of healthcare systems and the ART coverage within populations [10, 23, 24]. For example, Rwanda that has reached 96% ART coverage ranked lower in terms of PAFs than Congo, for example, which has a national ART coverage of 31% [13]. Throughout the entire spectrum of HIV infection, the risk of cancer progression remains high compared with HIV-negative populations, even in the early stages of HIV immunodeficiency [25, 26]. Although, several studies show the HPV-cancer risk increases as CD4 T cell counts fall, particularly below 200 mm^3^, emphasizing the importance of timely and comprehensive ART coverage [25, 27]. It is crucial for HPV vaccination to be included within a wider package of prevention tactics for PWHA irrespective of early or late immunodeficiency status.

Global HPV vaccine coverage remains low, with only 21% of girls and just 7% of boys vaccinated [14]. Only 2 of the 10 countries with the highest number of HIV-attributable cancers had single dose coverage rates above 50%. Various structural and behavioral barriers underlie this, including supply, financing, health-system capacity, acceptability, and vaccine hesitancy [28, 29]. Many countries are still recovering from the suspension of vaccines during the COVID-19 pandemic [30]. Over a quarter of the coverage of HPV vaccines achieved in 2019 was lost by 2021 [30]. The increase in antivaccine activism since the pandemic has added further challenges to vaccine uptake worldwide [31].

Our findings align with existing literature and highlight the need for a gender-neutral HPV vaccination. Research has shown that men who have sex with men (MSM) living with HIV are at a higher risk of anal cancer. Deshmukh et al reported an age-stratified SIR of 68.7 (95% CI 60.1–77.9) among men aged 15–59, nearly 3 times higher than our estimates [12]. Similarly, Clifford et al calculated an incidence rate of 85 (95% CI 82–89) [32]. Although, both estimates were limited to data from the US and Canada interventions targeting MSM communities in LMICs have shown variable outcomes, due to social stigma and prejudice, which limits access to healthcare [33]. Furthermore, female-only vaccination programs have provided limited herd immunity benefits [34]. The WHO recommends vaccinating boys where feasible and affordable, however only 30% of countries have introduced gender-neutral vaccination, including only 1 LMIC [35, 36]. Aside from reducing HPV infections, a gender-neutral approach promotes gender equity, minimizes vaccine-related stigma, and combats misinformation [37].

Integrating HPV vaccination with HIV services, as demonstrated with TB infection control and family planning [38, 39], would improve both vaccine coverage and cost efficiency [38]. However, in areas of high HIV prevalence, HPV vaccination at a young age- such as during childhood immunizations or upon secondary school entry- before potential exposure to high-risk HPV strains, would lead to a more substantial reduction in HPV-related cancers [29].

### Strengths and Limitations

This study re-evaluated the risk for developing 5 cancer subtypes in over 1.5 million PWHA. To our knowledge, we present the first global estimates of vulvar, vaginal, penile, and oropharyngeal cancer, which may be attributable to HIV worldwide. Sources of bias and heterogeneity were investigated using statistical and sensitivity analyses.

Nonetheless, our study has limitations. Meta-analyses of observational studies are limited by inherent discrepancies between populations and variable reporting on factors including ART coverage make them difficult to account for [32]. PWHA are more likely to use tobacco and alcohol, which are known risk factors of oropharyngeal cancer, making it harder to reach conclusive associations [40–42]. However, the inclusion of cohort studies, which adjusted for confounding factors improved the accuracy of our results.

The PAF generates a number representing the averted cancer cases, had the exposure, HIV, been omitted from the population calculation [18]. Considering the synergy between the 2 infections, HIV is not an independent risk factor; this could overestimate the PAFs in countries with high HIV prevalence [9, 43]. Without the research to deconstruct this interplay, this study provides the most accurate projections possible based on available data. Further, our methods extrapolate overall vaccine benefits for gender-neutral vaccination as included studies were not segregated by sex. The assumption that men will be covered by improved vaccine coverage via herd immunity may have overestimated our results, particularly as the burden of HIV among non-MSM populations vary between countries. Similarly, we included all cases of the 5 cancers, which may overestimate our results given that not all cases are caused by HPV infection [4]. The lack of data on histological subtypes within the IARC's database meant it was difficult to ascertain the true number.

Modeling assumptions were made during the vaccination analysis; a full description can be found in the appendix. Given the lack of data on HPV vaccine efficacy in PWHA, we chose simplified point estimates at 50%, 70%, and 90%. Similarly, the evidence on which strains drive noncervical cancers in PWHA is scarce. We assumed the bivalent vaccine to have wider protection abilities outside the primary strains, 16 and 18, which may overestimate our results. Where no data on national vaccine coverage could be sourced, the country was assumed to have no coverage. This was an underestimate, as some countries provide HPV vaccines to certain regions or privately, detailed in Supplementary Appendix Table 7. We also expect the incidence of cancer to be underestimated due to disparities in documentation of HPV-related cancers, worsened by the limits to definitive diagnoses for noncervical cancer globally [44]. Overall, only 2 nonhigh-income countries were included across our meta-analyses, limiting their extrapolation on a global context. For example, only North American studies were included for vulvar cancer. Lastly, we recognize that the parameters relied on global datasets; often data are poor quality and extrapolated from neighboring countries [1]. Our approximations should be interpreted tentatively within the context of these assumptions.

### Future Research

Despite the known increased risk of HPV acquisition and related cancers in PWHA, research into coinfection is scarce, particularly on noncervical cancers [4]. Data on noncervical cancer in PWHA in LMICs would valuable, particularly in areas of high prevalence. Our results demonstrate that further research recognizing the HPV vaccine's potential beyond cervical cancer is warranted, especially as screening programs are limited for these cancer types.

Multiple systematic reviews found a high vaccine immunogenicity and safety in PWHA but data from robust randomized controlled trials is lacking [21]. Research to aid future assessments of dosage and long-term protection in PWHA is needed [45]. The implications of dosage on cost and feasibility are particularly important for LMICs with high HIV prevalence [46]. Lastly, the public health benefits of vaccinating PWHA should be investigated, as the likelihood of HPV exposure prior to vaccination is high due to shared transmission routes [47]. Although difficult, studies could build upon our results for modeling HIV/HPV development through dynamic transmission models to further optimize vaccine strategies in PWHA.

## CONCLUSION

In conclusion, our results show that PWHA are at much greater risk of developing noncervical, HPV-related cancers, particularly anal cancer. For all subtypes, East Africa experienced the highest burden of HPV-related cancers attributable to HIV and AIDS. This region also has some of the lowest HPV vaccine coverage rates worldwide.

Concerted efforts to address the structural barriers to vaccine coverage and equity is vital. A gender-neutral HPV vaccine in childhood or adolescence, in countries with the highest number of HIV-attributable cancers, could escalate progress toward reversing the wide disparities in the distribution of HPV-related cancers worldwide.

## Supplementary Material

ofaf577_Supplementary_Data

## Supplementary Data

Supplementary materials are available at *Open Forum Infectious Diseases* online. Consisting of data provided by the authors to benefit the reader, the posted materials are not copyedited and are the sole responsibility of the authors, so questions or comments should be addressed to the corresponding author.
