# Supplementary material for: Global Estimates of the HPV-related, Noncervical Cancer Burden in People With HIV and AIDS and the Potential Effect of Improved HPV Vaccine Coverage in This Population: A Systematic Review, Meta-analysis and Modeling Study
Source: Open Forum Infect Dis. 2025 Sep 16;12(10):ofaf577. doi: 10.1093/ofid/ofaf577 (PMC12507086; doi:10.1093/ofid/ofaf577)
Supplement: ofaf577_Supplementary_Data [file ofaf577_supplementary_data.docx]

Appendix

***Table S1a:*** *EMBASE Search strategy*

| EMBASE: 2175 | | |
| --- | --- | --- |
| 1 | HIV.mp. or exp Human immunodeficiency virus/ | 510987 |
| 2 | Human immunodeficiency virus.mp. [mp=title, abstract, heading word, drug trade name, original title, device manufacturer, drug manufacturer, device trade name, keyword heading word, floating subheading word, candidate term word] | 522000 |
| 3 | AIDs.mp. [mp=title, abstract, heading word, drug trade name, original title, device manufacturer, drug manufacturer, device trade name, keyword heading word, floating subheading word, candidate term word] | 222001 |
| 4 | Acquired immunodeficiency syndrome.mp. or exp acquired immune deficiency syndrome/ | 581035 |
| 5 | 1 or 2 or 3 or 4 | 1082246 |
| 6 | Surveillance.mp. or exp monitoring/ | 1362756 |
| 7 | exp standardized incidence ratio/ or Incidence.mp. or incidence/ | 1651913 |
| 8 | Prevalence.mp. or exp prevalence/ | 1469702 |
| 9 | exp epidemiology/ or Epidemiolog*.mp. | 5645848 |
| 10 | exp sentinel surveillance/ or Sentinel.mp. | 60414 |
| 11 | Registr*.mp. | 550740 |
| 12 | exp cross-sectional study/ or Cross-sectional.mp. | 869481 |
| 13 | exp observational study/ or Observational.mp. | 560142 |
| 14 | exp cohort analysis/ or Cohort.mp. | 1716373 |
| 15 | Longitudinal study.mp. or exp longitudinal study/ | 234073 |
| 16 | Rapid assessment.mp. | 6198 |
| 17 | 6 or 7 or 8 or 9 or 10 or 11 or 12 or 13 or 14 or 15 or 16 | 8879012 |
| 18 | (Anal adj cancer).mp. [mp=title, abstract, heading word, drug trade name, original title, device manufacturer, drug manufacturer, device trade name, keyword heading word, floating subheading word, candidate term word] | 5282 |
|  |  |  |
| 20 | Anal intraepithelial neoplasia.mp. or exp anus carcinoma/ or exp anal intraepithelial neoplasia/ | 4676 |
| 21 | Anal malignancy.mp. | 28 |
| 22 | Anus tum?r.mp. [mp=title, abstract, heading word, drug trade name, original title, device manufacturer, drug manufacturer, device trade name, keyword heading word, floating subheading word, candidate term word] | 2290 |
| 23 | Anus carcinoma.mp. or exp anus carcinoma/ or exp anus tumor/ | 12834 |
| 24 | exp penis cancer/ or Peni* cancer.mp. | 10916 |
| 25 | Peni* Tum?r.mp. [mp=title, abstract, heading word, drug trade name, original title, device manufacturer, drug manufacturer, device trade name, keyword heading word, floating subheading word, candidate term word] | 2541 |
| 26 | Peni* Neoplasm.mp. [mp=title, abstract, heading word, drug trade name, original title, device manufacturer, drug manufacturer, device trade name, keyword heading word, floating subheading word, candidate term word] | 86 |
| 27 | Peni* Carcinoma.mp. [mp=title, abstract, heading word, drug trade name, original title, device manufacturer, drug manufacturer, device trade name, keyword heading word, floating subheading word, candidate term word] | 3006 |
| 28 | (Oropharyngeal adj cancer).mp. [mp=title, abstract, heading word, drug trade name, original title, device manufacturer, drug manufacturer, device trade name, keyword heading word, floating subheading word, candidate term word] | 6686 |
| 29 | Oropharynx tum?r.mp. [mp=title, abstract, heading word, drug trade name, original title, device manufacturer, drug manufacturer, device trade name, keyword heading word, floating subheading word, candidate term word] | 2334 |
| 30 | exp pharynx cancer/ or exp "head and neck cancer"/ or Pharyn* cancer.mp. | 259604 |
| 31 | Oropharynx squamous cell carcinoma.mp. or exp squamous cell carcinoma/ or exp oropharynx squamous cell carcinoma/ or exp oropharynx carcinoma/ | 232716 |
| 32 | Oropharynx carcinoma.mp. [mp=title, abstract, heading word, drug trade name, original title, device manufacturer, drug manufacturer, device trade name, keyword heading word, floating subheading word, candidate term word] | 4135 |
| 33 | (Head and neck).mp. [mp=title, abstract, heading word, drug trade name, original title, device manufacturer, drug manufacturer, device trade name, keyword heading word, floating subheading word, candidate term word] | 234091 |
| 34 | (Vagin* adj cancer).mp. [mp=title, abstract, heading word, drug trade name, original title, device manufacturer, drug manufacturer, device trade name, keyword heading word, floating subheading word, candidate term word] | 4170 |
| 35 | exp vagina carcinoma/ or Vagin* carcinoma.mp. | 9287 |
| 36 | Vagin* malignancies.mp. | 78 |
| 37 | Vagin* tum?r.mp. [mp=title, abstract, heading word, drug trade name, original title, device manufacturer, drug manufacturer, device trade name, keyword heading word, floating subheading word, candidate term word] | 2972 |
| 38 | (Vulv* adj cancer).mp. [mp=title, abstract, heading word, drug trade name, original title, device manufacturer, drug manufacturer, device trade name, keyword heading word, floating subheading word, candidate term word] | 7045 |
| 39 | exp vulva carcinoma/ or Vulv* carcinoma.mp. or exp vulvar squamous cell carcinoma/ | 8199 |
| 40 | (Vulv* adj malignanc*).mp. [mp=title, abstract, heading word, drug trade name, original title, device manufacturer, drug manufacturer, device trade name, keyword heading word, floating subheading word, candidate term word] | 322 |
| 41 | Vulv* tum?r.mp. [mp=title, abstract, heading word, drug trade name, original title, device manufacturer, drug manufacturer, device trade name, keyword heading word, floating subheading word, candidate term word] | 3799 |
| 42 | Human Papillomavirus.mp. or exp Wart virus/ | 78749 |
| 43 | HPV.mp. | 75146 |
| 44 | 42 or 43 | 96990 |
| 45 | 18 or 19 or 20 or 21 or 22 or 23 or 24 or 25 or 26 or 27 or 28 or 29 or 30 or 31 or 32 or 33 or 34 or 35 or 36 or 37 or 38 or 39 or 40 or 41 or 44 | 575800 |
| 46 | 5 and 17 and 45 and 49 | 2175 |

***Table S1b****: Global Health Search strategy*

| Global health: 434 | | |
| --- | --- | --- |
| 1 | HIV.mp. or exp human immunodeficiency viruses/ | 222234 |
| 2 | Human immunodeficiency virus.mp. | 196940 |
| 3 | AIDs.mp. or exp acquired immune deficiency syndrome/ | 85675 |
| 4 | Acquired immunodeficiency syndrome.mp. [mp=abstract, title, original title, heading words, cabicodes words] | 5445 |
| 5 | 1 or 2 or 3 or 4 | 232741 |
| 6 | exp surveillance/ or Surveillance.mp. or exp sentinel surveillance/ | 158243 |
| 7 | Incidence.mp. or exp incidence/ | 277903 |
| 8 | Prevalence.mp. [mp=abstract, title, original title, heading words, cabicodes words] | 403144 |
| 9 | epidemiology.sh. or Epidemiolog*.mp. | 485869 |
| 10 | Sentinel.mp. | 7697 |
| 11 | Registr*.mp. [mp=abstract, title, original title, heading words, cabicodes words] | 42616 |
|  |  |  |
| 13 | Observational.mp. or exp observational studies/ | 58988 |
| 14 | Cohort.mp. or exp cohort studies/ | 184564 |
| 15 | Longitudinal study.mp. or exp longitudinal studies/ | 143568 |
| 16 | Rapid assessment.mp. | 1339 |
| 17 | 6 or 7 or 8 or 9 or 10 or 11 or 12 or 13 or 14 or 15 or 16 | 1149541 |
| 18 | (Anal adj cancer).mp. [mp=abstract, title, original title, heading words, cabicodes words] | 1064 |
| 19 | (Anal adj dysplasia).mp. [mp=abstract, title, original title, heading words, cabicodes words] | 124 |
| 20 | Anal intraepithelial neoplasia.mp. or anal cancer.sh. | 740 |
| 21 | Anal malignancy.mp. | 5 |
| 22 | Peni* cancer.mp. or penile cancer.sh. | 399 |
| 23 | Peni* Tum?r.mp. [mp=abstract, title, original title, heading words, cabicodes words] | 5 |
| 24 | Peni* Carcinoma.mp. [mp=abstract, title, original title, heading words, cabicodes words] | 61 |
| 25 | (Oropharyngeal adj cancer).mp. [mp=abstract, title, original title, heading words, cabicodes words] | 1333 |
| 26 | Oropharynx tum?r.mp. [mp=abstract, title, original title, heading words, cabicodes words] | 1 |
| 27 | Oropharynx squamous cell carcinoma.mp. or oropharyngeal cancer.sh. or "head and neck cancer".sh. | 3373 |
| 28 | Oropharynx carcinoma.mp. | 12 |
| 29 | (Head and neck).mp. [mp=abstract, title, original title, heading words, cabicodes words] | 7652 |
| 30 | (Vagin* adj cancer).mp. [mp=abstract, title, original title, heading words, cabicodes words] | 224 |
| 31 | Vagin* carcinoma.mp. [mp=abstract, title, original title, heading words, cabicodes words] | 17 |
| 32 | Vagin* malignancies.mp. [mp=abstract, title, original title, heading words, cabicodes words] | 4 |
| 33 | Vagin* tum?r.mp. [mp=abstract, title, original title, heading words, cabicodes words] | 1 |
| 34 | (Vulv* adj cancer).mp. [mp=abstract, title, original title, heading words, cabicodes words] | 363 |
| 35 | Vulv* carcinoma.mp. [mp=abstract, title, original title, heading words, cabicodes words] | 48 |
| 36 | (Vulv* adj malignanc*).mp. [mp=abstract, title, original title, heading words, cabicodes words] | 9 |
| 37 | Vulv* tum?r.mp. [mp=abstract, title, original title, heading words, cabicodes words] | 5 |
| 38 | Human Papillomavirus.mp. or exp human papillomaviruses/ | 25113 |
| 39 | HPV.mp. [mp=abstract, title, original title, heading words, cabicodes words] | 22279 |
| 40 | Wart virus.mp. or Papillomaviridae.od. or human papillomaviruses.sh. | 24029 |
| 41 | HPV infection.mp. [mp=abstract, title, original title, heading words, cabicodes words] | 7293 |
| 42 | 18 or 19 or 20 or 21 or 22 or 23 or 24 or 25 or 26 or 27 or 28 or 29 or 30 or 31 or 32 or 33 or 34 or 35 or 36 or 37 or 38 or 39 or 40 or 41 or 42 | 10284 |
| 43 | 5 and 17 and 38 and 42 | 434 |

***Table S1c****: Medline Search strategy*

| MEDLINE: 1137 results | | |
| --- | --- | --- |
| 1 | exp HIV/ or HIV.mp. or exp HIV Infections/ or exp HIV-1/ | 453302 |
| 2 | Human immunodeficiency virus.mp. | 110262 |
|  |  |  |
| 4 | Acquired immunodeficiency syndrome.mp. [mp=title, book title, abstract, original title, name of substance word, subject heading word, floating sub-heading word, keyword heading word, organism supplementary concept word, protocol supplementary concept word, rare disease supplementary concept word, unique identifier, synonyms, population supplementary concept word, anatomy supplementary concept word] | 93763 |
| 5 | 1 or 2 or 3 or 4 | 534790 |
| 6 | Surveillance.mp. [mp=title, book title, abstract, original title, name of substance word, subject heading word, floating sub-heading word, keyword heading word, organism supplementary concept word, protocol supplementary concept word, rare disease supplementary concept word, unique identifier, synonyms, population supplementary concept word, anatomy supplementary concept word] | 301672 |
| 7 | Incidence.mp. [mp=title, book title, abstract, original title, name of substance word, subject heading word, floating sub-heading word, keyword heading word, organism supplementary concept word, protocol supplementary concept word, rare disease supplementary concept word, unique identifier, synonyms, population supplementary concept word, anatomy supplementary concept word] | 1064876 |
| 8 | exp Incidence/ | 305446 |
| 9 | exp Prevalence/ or Prevalence.mp. | 922769 |
| 10 | exp Epidemiological Monitoring/ or exp Epidemiology/ or Epidemiolog*.mp. | 2468763 |
| 11 | Sentinel.mp. or exp Sentinel Surveillance/ | 41103 |
| 12 | Registr*.mp. | 426057 |
| 13 | exp Cross-Sectional Studies/ or Cross-sectional.mp. | 695378 |
| 14 | Observational.mp. or exp Observational Study/ | 342901 |
| 15 | Cohort.mp. or exp Cohort Studies/ | 2941449 |
| 16 | Longitudinal study.mp. or exp Longitudinal Studies/ | 196383 |
| 17 | Rapid assessment.mp. | 4556 |
| 18 | 6 or 7 or 8 or 9 or 10 or 11 or 12 or 13 or 14 or 15 or 16 or 17 | 6227603 |
| 19 | (Anal adj cancer).mp. [mp=title, book title, abstract, original title, name of substance word, subject heading word, floating sub-heading word, keyword heading word, organism supplementary concept word, protocol supplementary concept word, rare disease supplementary concept word, unique identifier, synonyms, population supplementary concept word, anatomy supplementary concept word] | 3030 |
| 20 | (Anal adj dysplasia).mp. [mp=title, book title, abstract, original title, name of substance word, subject heading word, floating sub-heading word, keyword heading word, organism supplementary concept word, protocol supplementary concept word, rare disease supplementary concept word, unique identifier, synonyms, population supplementary concept word, anatomy supplementary concept word] | 281 |
| 21 | Anal intraepithelial neoplasia.mp. | 544 |
| 22 | exp Anus Neoplasms/ or Anal malignancy.mp. | 7335 |
| 23 | Anus tum?r.mp. [mp=title, book title, abstract, original title, name of substance word, subject heading word, floating sub-heading word, keyword heading word, organism supplementary concept word, protocol supplementary concept word, rare disease supplementary concept word, unique identifier, synonyms, population supplementary concept word, anatomy supplementary concept word] | 4 |
|  |  |  |
| 25 | exp Penile Neoplasms/ or Peni* cancer.mp. | 6725 |
| 26 | Peni* Tum?r.mp. [mp=title, book title, abstract, original title, name of substance word, subject heading word, floating sub-heading word, keyword heading word, organism supplementary concept word, protocol supplementary concept word, rare disease supplementary concept word, unique identifier, synonyms, population supplementary concept word, anatomy supplementary concept word] | 102 |
| 27 | Peni* Neoplasm.mp. | 43 |
| 28 | Peni* Carcinoma.mp. | 791 |
| 29 | (Oropharyngeal adj cancer).mp. [mp=title, book title, abstract, original title, name of substance word, subject heading word, floating sub-heading word, keyword heading word, organism supplementary concept word, protocol supplementary concept word, rare disease supplementary concept word, unique identifier, synonyms, population supplementary concept word, anatomy supplementary concept word] | 4563 |
| 30 | Oropharynx tum?r.mp. [mp=title, book title, abstract, original title, name of substance word, subject heading word, floating sub-heading word, keyword heading word, organism supplementary concept word, protocol supplementary concept word, rare disease supplementary concept word, unique identifier, synonyms, population supplementary concept word, anatomy supplementary concept word] | 9 |
| 31 | Pharyn* cancer.mp. [mp=title, book title, abstract, original title, name of substance word, subject heading word, floating sub-heading word, keyword heading word, organism supplementary concept word, protocol supplementary concept word, rare disease supplementary concept word, unique identifier, synonyms, population supplementary concept word, anatomy supplementary concept word] | 1103 |
| 32 | exp Carcinoma, Squamous Cell/ or exp Oropharyngeal Neoplasms/ or exp "Squamous Cell Carcinoma of Head and Neck"/ or Oropharynx squamous cell carcinoma.mp. or exp "Head and Neck Neoplasms"/ | 425875 |
| 33 | (Head and neck).mp. [mp=title, book title, abstract, original title, name of substance word, subject heading word, floating sub-heading word, keyword heading word, organism supplementary concept word, protocol supplementary concept word, rare disease supplementary concept word, unique identifier, synonyms, population supplementary concept word, anatomy supplementary concept word] | 152147 |
| 34 | (Vagin* adj cancer).mp. [mp=title, book title, abstract, original title, name of substance word, subject heading word, floating sub-heading word, keyword heading word, organism supplementary concept word, protocol supplementary concept word, rare disease supplementary concept word, unique identifier, synonyms, population supplementary concept word, anatomy supplementary concept word] | 920 |
| 35 | exp Vaginal Neoplasms/ or Vagin* carcinoma.mp. | 5597 |
| 36 | Vagin* malignancies.mp. | 56 |
| 37 | Vagin* tum?r.mp. [mp=title, book title, abstract, original title, name of substance word, subject heading word, floating sub-heading word, keyword heading word, organism supplementary concept word, protocol supplementary concept word, rare disease supplementary concept word, unique identifier, synonyms, population supplementary concept word, anatomy supplementary concept word] | 149 |
| 38 | (Vulv* adj cancer).mp. [mp=title, book title, abstract, original title, name of substance word, subject heading word, floating sub-heading word, keyword heading word, organism supplementary concept word, protocol supplementary concept word, rare disease supplementary concept word, unique identifier, synonyms, population supplementary concept word, anatomy supplementary concept word] | 2436 |
|  |  |  |
|  |  |  |
| 41 | Vulv* tum?r.mp. [mp=title, book title, abstract, original title, name of substance word, subject heading word, floating sub-heading word, keyword heading word, organism supplementary concept word, protocol supplementary concept word, rare disease supplementary concept word, unique identifier, synonyms, population supplementary concept word, anatomy supplementary concept word] | 140 |
| 42 | HPV.mp. or exp Papillomavirus Infections/ or exp Papillomaviridae/ | 74135 |
| 43 | Human Papillomavirus.mp. [mp=title, book title, abstract, original title, name of substance word, subject heading word, floating sub-heading word, keyword heading word, organism supplementary concept word, protocol supplementary concept word, rare disease supplementary concept word, unique identifier, synonyms, population supplementary concept word, anatomy supplementary concept word] | 48227 |
| 44 | Wart virus.mp. | 127 |
| 45 | HPV infection.mp. [mp=title, book title, abstract, original title, name of substance word, subject heading word, floating sub-heading word, keyword heading word, organism supplementary concept word, protocol supplementary concept word, rare disease supplementary concept word, unique identifier, synonyms, population supplementary concept word, anatomy supplementary concept word] | 13696 |
| 46 | 19 or 20 or 21 or 22 or 23 or 24 or 25 or 26 or 27 or 28 or 29 or 30 or 31 or 32 or 33 or 34 or 35 or 36 or 37 or 38 or 39 or 40 or 41 or 42 or 43 or 44 or 45 | 512379 |
| 47 | 5 and 18 and 46 | 1137 |

| Inclusion | Exclusion |
| --- | --- |
| Observational studies of adults living with HIV or AIDs | Reports outcomes on cervical cancer, HPV infection, pre-cursor lesions or groups outcomes e.g. anogenital |
| Reports the risk of developing a HPV-related, non-cervical cancer as a risk ratio (RR), incidence rate ratio (IRR), or standardised incidence ratio (SIR) | Self-reported HIV status |
| Comparison with a HIV negative control group or the general reference population | ≤1 case of cancer in PWHA in the study |
| Published in a peer-reviewed source |  |
| Cases defined by International Classification of Diseases 10^th^ edition [ICD-10] |  |

**Table S2:** Predefined inclusion and exclusion criteria

**Description of methods and assumptions**

*Step 1:*

We conducted a systematic literature review and meta-analysis to generate a pooled risk ratio comparing PWHA to HIV negative populations for developing anal, oropharyngeal, penile, vaginal, vulvar cancer respectively. We carried out a random effects model considering the high heterogeneity when meta-analysing observational studies due to difference in study design, population, geographical differences.

*Step 2:*

Risk ratios were extracted for the risk of developing each cancer for PWHA compared to HIV negative people from included studies. This was pooled using the generic inverse variance method in the ‘meta’ package on R.

*Step 3:*

UNAIDS HIV prevalence for 2022 for 160 countries was collected onto Microsoft Excel along with incident cancer cases for each cancer in 2022 from GLOBOCAN for 185 countries.

*Step 4:*

*Assumptions:*

- We assumed that risk estimates, risk ratios, standardised incidence ratios, incidence rate ratios were equivalent given the rare outcomes
- We assumed our pooled risk ratio would be transferable to LMICs
- We assumed each study was independent and therefore estimates were limited by residual confounding
- HIV is completely eliminable and independent risk factor

*Step 5:*

We calculated the PAF (attributable to HIV and AIDs) using the following equation:


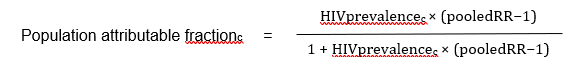


We multiplied the PAF to HIV and AIDs by the number of incident cancer cases for each country in IARC’s 2022 data for the number of cases attributable to HIV/AIDs.

*PAF_c_  X Number of cancer cases_c_ = Number of cancer cases attributable to HIV*

***Figure S1****: Flowchart for calculations for South Africa*

*Step 6:*

We divided this number into two using vaccine coverage data for each specific country from the WHO database. We then divided this using efficacy estimates of 50%, 70%, 90% to get the potential number of cancers averted.

*Assumptions:*

- The efficacy estimates are reflective of the true efficacy in PWHA
- The vaccine covers all HPV strains that lead to development of non-cervical cancers
- 100% of cancer cases diagnosed in the IARC’s database are linked to HPV, and can be prevented by the vaccine
- Countries with 0 coverage assumed to have no programme if cross checked with HPV world data
- Countries with 0 HIV prevalence removed from our analysis
- No sex aggregation occurred. Risks vary depending on the sex however this was not accounted for. Data on vaccine efficacy in men is limited.
- We used a static transmission model using retrospective data which cannot account for temporal associations
- We assumed that the WHO’s coverage levels extended to men via herd immunity mechanisms.

***NUMBER OF CANCER CASES ATTRIBUTABLE TO HIV***

***CANCER CASES IN VACCINATED***

**VACCINE COVERAGE**

**VACCINE EFFICACY**

**CANCER CASES PREVENTED**

**410**

**246**

90%

60%

**221**

**410 X 0.6**

**246 X 0.9**

**Table S3**: Baseline characteristics of included studies

| Study, year | Study design | Study period | Country | Comparator population | Number of HIV positive | Mean age (HIV positive) | Ethnicity (HIV positive) | ART access (%) | Female (%) |
| --- | --- | --- | --- | --- | --- | --- | --- | --- | --- |
| Mayor AM. et al, 2016 ^1^ | Registry linkage study | 1991-2010 | Puerto Rico | Puerto Rican general population | 826 | 41.5 | Hispanic | 59.2% | 37.90% |
| Van Leeuwen MT. et al, 2009^2^ | Registry linkage study | 1982-2004 | Australia | Australian general population | 14,019 | - | - | - | 7% |
| Wong IK.J. et al, 2022^3^ | Registry linkage study | 1982-2012 | Australia | Australian general population | 28,703 | 48.5 | - | - | 9.80% |
| Hessol NA. et al, 2018^4^ | Registry linkage study | 1990-2010 | USA | SEER data registry | 22 623 | - | 71% Caucasian, 11% Black, 13% Hispanic 4% Other | - | 3% |
| Chen M. et al, 2014^5^ | Registry linkage study | 1998-2009 | Taiwan | Taiwan general population | 15,269 | - | - | - | 10.8% |
| Franceschi S. et al, 2010^6^ | Registry linkage study | 1985-2007 | Switzerland | Swiss general population | 9429 | - | - | - | 29% |
| Hernandez-Ramirez RU. et al, 2017^7^ | Registry linkage study | 1996-2012 | USA | USA general population | 448,258 | - | 51% Black, 28% Hispanic, 21% Caucasian | - | 30.2% |
| Salters KA. et al, 2016^8^ | Registry linkage study | 1994-2008 | Canada | Canadian general population | 2211 | - | 16% Caucasian, 83% Other | 62% adherent | 100% |
| Godbole SV. et al, 2016^9^ | Registry linkage study | 1996-2008 | India | Indian general population | 32,575 | - | - | - | 35.90% |
| Seaberg EC. et al, 2010^10^ | Registry linkage study | 1984-2007 | USA | USA general population | 2918 | - | 73% Caucasian | - | 0% |
| Maso LD. et al, 2009^11^ | Registry linkage study | 1986-2004 | Italy | Italian general population | 21951 | - | - | - | 22% |
| Chaturvedi AK et al, 2009^12^ | Registry linkage study | 1996-2004 | USA | USA general population | 185781 | - | 47.1% Black, 21.1% Hispanic, 29.2% Caucasian | - | 24.80% |
| Engels EA et al, 2006^13^ | Registry linkage study | 1997-2004 | USA | USA general population | 104417 | - | - | - | 25.4% |
| Tanaka LF et al, 2018^14^ | Registry linkage study | 1997-2012 | Brazil | Brazilian general population | 2000 | - | 55.6% Caucasian, 20.6% non-white | - | 27% |
| Piketty C. et al, 2012^15^ | Cohort study | 1992-2008 | France | French general population | 109,771 | - | - | 89.40% | - |
| Serraino D. et al, 2007^16^ | Cohort study | 1988-2004 | France | French general population | 8074 | - | - | 25% | 28.8% |
| Newnham A. et al, 2004^17^ | Cohort study | 1985-2001 | England | England general population | 33,190 | - | - | - | 27.2% |
| Beachler DC. et al, 2014^18^ | Cohort study | 1996-2009 | USA | USA general population | 82,375 | - | 38% Black, 45% White, 17% Other | 20% | 14% |
| Raffetti E. et al, 2015^19^ | Cohort study | 1986-2012 | Italy | Italian general population | 16,268 | 34.2 | - | 87.2% | 25.5% |
| Zhu W. et al, 2019^20^ | Cohort study | 2008-2011 | China | Chinese general population | 399,451 | - | - |  | 30.2% |
| Lee SO. et al, 2022^21^ | Cohort study | 2004-2017 | Korea | Korean general population | 11,737 | - | - | 94% | 7% |
| Silverberg MJ. et al, 2009^22^ | Cohort study | 1996-2007 | USA | Matched HIV-negative controls | 20,277 | 40.9 | 3.9% Asian, 18.9% Black, 20.8% Hispanic, 55.6% Caucasian | 27.3% | 9.5% |
| Engsig FN. et al, 2011^23^ | Cohort | 1995-2009 | Denmark | Matched HIV negative controls | 5053 | - | 78.7% Caucasians | - | 24.3% |
| Clark E. et al, 2021^24^ | Cohort | 1999-2016 | USA | Matched HIV negative controls | 1454 | - | 22.2% Caucasian, 64.2% Black | - | 100% |
| Park LS. et al, 2016^25^ | Cohort | 1997-2012 | USA | Matched HIV negative controls | 44, 784 | - | 39% Caucasian, 49% Black, 7% Hispanic | - | 2% |

***Table 4:*** *Summary table of studies included for each cancer*

| **Vaginal cancer** | | | | | | | | |
| --- | --- | --- | --- | --- | --- | --- | --- | --- |
| **Study, year** | **Study design** | **Country** | **Study period** | **Number of PWHA** | **Cancer cases** | **RR** | **CI 95%** |  |
| **Mayor AM. et al, 2016** | Registry linkage | Puerto Rico | 2003-2009 | 826 | 2 | 40 | (5-144) |  |
| **Hernandez-Ramirez RU et al, 2017** | Registry linkage | USA | 1996-2012 | 448,258 | 25 | 3.74 | (2.42-5.52) |  |
| **Godbole SV et al, 2016** | Registry linkage | India | 1996-2008 | 32,575 | 2 | 49 | (5.9-177) |  |
| **Clark E et al,, 2021** | Cohort | USA | 1996-2012 | 1454 | 2 | 9.89 | (1.4-70.8) |  |

***Table S4a:*** *Summary table of study characteristics and results for* ***vaginal*** *cancer*

| **Anal cancer** | | | | | | | |
| --- | --- | --- | --- | --- | --- | --- | --- |
| **Study, year** | **Study design** | **Country** | **Study period** | **Number of PWHA** | **Cancer cases** | **RR** | **CI 95%** |
| **Mayor AM. et al, 2016** | Registry linkage | Puerto Rico | 2003-2009 | 826 | 3 | 18 | (4-52) |
| **Hessol NA et al, 2018** | Registry linkage | USA | 1990-2010 | 22,623 | 173 | 46.6 | (39.7-53.6) |
| **Franceschi S et al, 2010** | Registry linkage | Switzerland | 2002-2006 | 9429 | 6 | 49.9 | (18-109) |
| **Wong IK.J et al, 2022** | Registry linkage | Australia | 1982-2012 | 28,703 | 54 | 37.71 | (28.33-49.21) |
| **Hernandez-Ramirez RU et al, 2017** | Registry linkage | USA | 1996-2012 | 448,258 | 1568 | 20.2 | (19.2-21.2) |
| **Raffetti E et al, 2015** | Cohort | Italy | 1986-2012 | 16,268 | 24 | 3.1 | (2-4.7) |
| **Seaberg EC. et al, 2010** | Registry linkage | USA | 1984-2007 | 2918 | 15 | 13.5 | (3.01-126.4) |
| **Park LS. et al, 2016** | Cohort | USA | 2009-2012 | 44,784 | 76 | 77 | (28-218) |
| **Serraino D et al, 2007** | Cohort | France | 1988-2004 | 8074 | 5 | 33 | (11-76) |
| **Silverberg MJ et al, 2009** | Cohort | USA | 1996-2007 | 20,277 | 110 | 101 | (54.3-189.9) |
| **Newnham A et al, 2004** | Cohort | England | 1985-2001 | 33,190 | 18 | 23.1 | (13.7-36.5) |
| **Piketty C et al, 2012** | Cohort | France | 2005-2008 | 109,771 | 106 | 50.2 | (41.1-60.8) |
| **Lee SO. et al, 2022** | Cohort | Korea | 2004-2017 | 11,737 | 19 | 69.52 | (41.84-108.57) |
| **Maso LD et al, 2009** | Registry linkage | Italy | 1986-2004 | 21951 | 11 | 44 | 21.8-78.9 |
| **Engels EA et al, 2006** | Registry linkage | USA | 1997-2004 | 104417 | 43 | 19.6 | 14.2-26.4 |
| **Van Leeuwen MT et al, 2009** | Registry linkage | Australia | 2000-2004 | 14,019 | 19 | 32.11 | 19.33-50.14 |
| **Chen M. et al, 2014** | Registry linakge | Taiwan | 1998-2009 | 15269 | 29 | 19.1 | 12.80-27.50 |

***Table S4b****: Summary table of study characteristics and results for* ***anal*** *cancer*

| **Penile cancer** | | | | | | | |
| --- | --- | --- | --- | --- | --- | --- | --- |
| **Study, year** | **Study type** | **Country** | **Study period** | **Number of PWHA** | **Cancer cases** | **RR** | **CI 95%** |
| **Hessol NA. et al, 2018** | Registry linkage | USA | 1990-2010 | 22,623 | 10 | 3.8 | (1.4-6.1) |
| **Godbole SV et al, 2016** | Registry linkage | India | 1996-2008 | 32,575 | 5 | 15.6 | (4.3-40) |
| **Hernandez-Ramirez RU et al, 2017** | Registry linkage | USA | 1996-2012 | 448,258 | 114 | 5.68 | (4.68-6.82) |
| **Raffetti E. et al, 2015** | Cohort | Italy | 1986-2012 | 16,268 | 8 | 28 | (12.1-55.2) |
| **Park LS. et al, 2016** | Cohort | USA | 2009-2012 | 44,784 | 3 | 5.8 | (0.46-73) |
| **Silverberg MJ. et al, 2009** | Cohort | USA | 1996-2007 | 20,277 | 4 | 5.8 | (1.7-19.8) |
| **Zhu W et al, 2019** | Cohort | China | 2008-2011 | 399,451 | 6 | 2.8 | (1-6.1) |
| **Maso LD et al, 2009** | Registry linkage | Italy | 1986-2005 | 21951 | 3 | 12 | (2.35-35.5) |
| **Chaturvedi AK et al, 2009** | Registry linkage | USA | 1996-2004 | 185781 | 20 | 5.3 | (3.26-8.61) |
| **Engels EA et al, 2006** | Registry linkage | USA | 1997-2004 | 104417 | 4 | 8 | (2.2-20.6) |
| **Tanaka LF et al, 2018** | Registry linkage | Brazil | 1997-2012 | 2000 | 6 | 1.77 | (0.80-3.94) |

**Table S4c:** Summary table of study characteristics and results for penile cancer

| **Vulvar cancer** | | | | | | | |
| --- | --- | --- | --- | --- | --- | --- | --- |
| **Study, year** | **Study design** | **Country** | **Follow up period** | **Number of PWHA** | **Cancer cases** | **RR** | **CI 95%** |
| **Hessol NA. et al, 2018** | Registry linkage | USA | 1990-2010 | 22,623 | 13 | 13.34 | (6.09-20.59) |
| **Hernandez-Ramirez RU. et al, 2017** | Registry linkage | USA | 1996-2012 | 448,258 | 151 | 9.82 | (8.31-11.5) |
| **Salters KA. et al, 2016** | Registry linkage | Canada | 1994-2008 | 2211 | 2 | 5.88 | (0.71-21.25) |
| **Clark E et al, 2021** | Cohort | USA | 1996-2012 | 1454 | 2 | 2.13 | (3-13.4) |

***Table S4d:*** *Summary table of study characteristics and results for* ***vulvar*** *cancer*

| **Oropharyngeal cancers** | | | | | | | |
| --- | --- | --- | --- | --- | --- | --- | --- |
| **Study, year** | **Study type** | **Country** | **Study period** | **Number of PWHA** | **Cancer cases** | **RR** | **95% CI** |
| **Seaberg EC. et al, 2010** | Registry linkage | USA | 1984-2007 | 2918 | 5 | 2.64 | (0.5-17.3) |
| **Beachler DC. et al, 2014** | Cohort | USA | 1996-2009 | 82,375 | 66 | 3.2 | (2.5-4.1) |
| **Newnham A et al, 2004** | Cohort | England | 1985-2001 | 33,190 | 4 | 5 | (1.4-12.8) |
| **Engsig FN et al, 2011** | Cohort | Denmark | 1995-2009 | 5053 | 16 | 2.96 | (1.72-5.09) |
| **Silverberg MJ. et al, 2009** | Cohort | USA | 1996-2007 | 20,277 | 11 | 2 | (1.1-3.9) |
| **Park LS. et al, 2016** | Cohort | USA | 2009-2012 | 44,784 | 29 | 1.7 | (1-2.9) |
| **Lee SO. et al, 2022** | Cohort | Korea | 2004-2017 | 11,737 | 11 | 2.97 | (1.48-5.32) |
| **Mayor AM. et al, 2016** | Registry linkage | Puerto Rico | 2003-2009 | 826 | 6 | 3.4 | (1.2-7) |
| **Van Leeuwen MT. et al, 2009** | Registry linkage | Australia | 2000-2004 | 14,019 | 7 | 1.65 | (0.66-3.39) |
| **Chen M et al, 2014** | Registry linkage | Taiwan | 1998-2009 | 15,269 | 19 | 5.4 | (3.25-8.42) |
| **Hernandez-**  **Ramirez RU. et al, 2017** | Registry linkage | USA | 1996-2012 | 448,258 | 297 | 1.75 | (1.56-1.97) |
| **Godbole SV. et al, 2016** | Registry linkage | India | 1996-2008 | 32,575 | 5 | 9.9 | (3.2-23.2) |
| **Chaturvedi AK. et al, 2009** | Registry linkage | USA | 1996-2004 | 185781 | 59 | 1.6 | (1.2-2.1) |
| **Engels EA. et al, 2006** | Registry linkage | USA | 1997-2004 | 104417 | 31 | 2.10 | (1.4-3.0) |

***Table S4e:*** *Summary table of study characteristics and results for* ***oropharyngeal*** *cancer*

***Appendix 1: References of included studies***

1. Mayor AM, Santiago-Rodriguez EJ, Rios-Olivares E, Tortolero-Luna G, Hunter-Mellado RF. Malignancies trends in a hispanic cohort of HIV persons in puerto rico before and after cART. Int J Cancer Res. 2016 Mar 15;12(2):92–100.

2. Van Leeuwen MT, Vajdic CM, Middleton MG, McDonald AM, Law M, Kaldor JM, et al. Continuing declines in some but not all HIV-associated cancers in Australia after widespread use of antiretroviral therapy. AIDS. 2009;23(16):2183–90.

3. Wong IKJ, Grulich AE, Poynten IM, Polizzotto MN, van Leeuwen MT, Amin J, et al. Time trends in cancer incidence in Australian people living with HIV between 1982 and 2012. HIV Med. 2022 Feb 1;23(2):134–45.

4. Hessol NA, Whittemore H, Vittinghoff E, Hsu LC, Ma D, Scheer S, et al. Incidence of first and second primary cancers diagnosed among people with HIV, 1985–2013: a population-based, registry linkage study. Lancet HIV. 2018 Nov 1;5(11):e647–55.

5. Chen M, Jen I, Chen YH, Lin MW, Bhatia K, Sharp GB, et al. Cancer Incidence in a Nationwide HIV/AIDS Patient Cohort in Taiwan in 1998-2009. JAIDS Journal of Acquired Immune Deficiency Syndromes 65(4):p 463-472, April 1, 2014. | DOI: 10.1097/QAI.0000000000000065EPI.

6. Franceschi S, Lise M, Clifford GM, Rickenbach M, Levi F, Maspoli M, et al. Changing patterns of cancer incidence in the early-and late-HAART periods: The Swiss HIV Cohort Study. Br J Cancer. 2010 Jul 27;103(3):416–22.

7. Hernández-Ramírez RU, Shiels MS, Dubrow R, Engels EA. Cancer risk in HIV-infected people in the USA from 1996 to 2012: a population-based, registry-linkage study. Lancet HIV. 2017 Nov 1;4(11):e495–504.

8. Salters KA, Cescon A, Zhang W, Ogilvie G, Murray MCM, Coldman A, et al. Cancer incidence among HIV-positive women in British Columbia, Canada: Heightened risk of virus-related malignancies. HIV Med. 2016 Mar 1;17(3):188–95.

9. Godbole S V., Nandy K, Gauniyal M, Nalawade P, Sane S, Koyande S, et al. HIV and cancer registry linkage identifies a substantial burden of cancers in persons with HIV in India. Medicine (United States). 2016;95(37).

10. Seaberg EC, Wiley D, Martínez-Maza O, Chmiel JS, Kingsley L, Tang Y, et al. Cancer incidence in the multicenter aids cohort study before and during the HAART era: 1984 to 2007. Cancer. 2010 Dec 1;116(23):5507–16.

11. Dal Maso L, Polesel J, Serraino D, Lise M, Piselli P, Falcini F, et al. Pattern of cancer risk in persons with AIDS in Italy in the HAART era. Br J Cancer. 2009 Mar 10;100(5):840–7.

12. Chaturvedi AK, Madeleine MM, Biggar RJ, Engels EA. Risk of Human Papillomavirus–Associated Cancers Among Persons With AIDS. JNCI: Journal of the National Cancer Institute. 2009 Aug 19;101(16):1120–30.

13. Engels EA, Pfeiffer RM, Goedert JJ, Virgo P, Mcneel TS, Scoppa SM, et al. Trends in cancer risk among people with AIDS in the United States 1980-2002.

14. Tanaka LF, Latorre MRDO, Gutierrez EB, Curado MP, Froeschl G, Heumann C, et al. Risk for cancer among people living with AIDS, 1997–2012: the São Paulo AIDS–cancer linkage study. European Journal of Cancer Prevention. 2018 Jul;27(4):411–7.

15. Piketty C, Selinger-Leneman H, Bouvier AM, Belot A, Mary-Krause M, Duvivier C, et al. Incidence of HIV-related anal cancer remains increased despite long-term combined antiretroviral treatment: Results from the french hospital database on HIV. Journal of Clinical Oncology. 2012 Dec 10;30(35):4360–6.

16. Serraino D, Piselli P, Busnach G, Burra P, Citterio F, Arbustini E, et al. Risk of cancer following immunosuppression in organ transplant recipients and in HIV-positive individuals in southern Europe. Eur J Cancer. 2007 Sep;43(14):2117–23.

17. Newnham A, Harris J, Evans HS, Evans BG, Møller H. The risk of cancer in HIV-infected people in southeast England: A cohort study. Br J Cancer. 2005 Jan 17;92(1):194–200.

18. Beachler DC, Abraham AG, Silverberg MJ, Jing Y, Fakhry C, Gill MJ, et al. Incidence and risk factors of HPV-related and HPV-unrelated Head and Neck Squamous Cell Carcinoma in HIV-infected individuals. Oral Oncol. 2014 Dec 1;50(12):1169–76.

19. Raffetti E, Albini L, Gotti D, Segala D, Maggiolo F, Di Filippo E, et al. Cancer incidence and mortality for all causes in HIV-infected patients over a quarter century: A multicentre cohort study Disease epidemiology - Infectious. BMC Public Health. 2015;15(1).

20. Zhu W, Mao Y, Tang H, McGoogan JM, Zhang ZF, Detels R, et al. Spectrum of malignancies among the population of adults living with HIV infection in China: A nationwide follow-up study, 2008–2011. PLoS One. 2019 Jul 1;14(7).

21. Lee SO, Lee JE, Lee S, Lee SH, Kang JS, Son H, et al. Nationwide population-based incidence of cancer among patients with HIV/AIDS in South Korea. Sci Rep. 2022 Dec 1;12(1).

22. Silverberg MJ, Chao C, Leyden WA, Xu L, Tang B, Horberg MA, et al. HIV infection and the risk of cancers with and without a known infectious cause. AIDS. 2009 Nov;23(17):2337–45.

23. Engsig FN, Gerstoft J, Kronborg G, Larsen CS, Pedersen G, Pedersen C, et al. Head and neck cancer in HIV patients and their parents: A Danish cohort study. Clin Epidemiol. 2011 Jul 21;3(1):217–27.

24. Clark E, Chen L, Dong Y, Raychaudhury S, White D, Kramer JR, et al. Veteran Women Living With Human Immunodeficiency Virus Have Increased Risk of Human Papillomavirus (HPV)-Associated Genital Tract Cancers. Clinical Infectious Diseases. 2021 May 1;72(9):E359–66.

25. Park LS, Tate JP, Sigel K, Rimland D, Crothers K, Gibert C, et al. Time trends in cancer incidence in persons living with HIV/AIDS in the antiretroviral therapy era: 1997-2012. Vol. 30, AIDS. Lippincott Williams and Wilkins; 2016. p. 1795–806.

***Table S5:*** *Newcastle-Ottawa risk of bias tool for all included studies. > 7 = low risk of bias. 6 = medium risk of bias. 5 = high risk of bias.*

|  | S1 - selection | | | | S2 - Comparability | S3 - Outcome | | | Rating |
| --- | --- | --- | --- | --- | --- | --- | --- | --- | --- |
| **Study** | S1a - Representativeness of the exposed cohort  S1b - Selection of non-exposed cohort  S1c - Ascertainment of exposure  S1d – Demonstrate that outcome of interest was not present at start of the study | | | | S2 – Comparability of cohorts based on design or analysis | S3a – Assessment of outcomes  S3b – Long enough follow-up?  S3c – Adequacy of follow up of cohorts | | |  |
|  |  |  |  |  | **C1** | **O1** | **O2** | **O3** | **Rating** |
| **Mayor AM. et al, 2016** | * | * | * | * | ***** | ***** | ***** |  | **7** |
| **Van Leeuwen MT. et al, 2009** | * | * | * | * | * | * | * | NA | **7** |
| **Raffetti E et al, 2015** | * | * | * | * | * | * | * | NA | **7** |
| **Wong IKJ. et al, 2021** | * | * | * |  | * | * | * |  | **7** |
| **Beachler DC. et al, 2014** | * | * | * | * | * | * | * | * | **7** |
| **Hessol NA. et al, 2018** | * | * | * |  | * | * |  |  | **5** |
| **Newnham A et al, 2004** | * | * | * | * | * | * | * |  | **7** |
| **Chen M et al, 2014** | * | * | * |  | * | * | * |  | **7** |
| **Franceschi S et al, 2010** | * | * | * | * | * | * | * |  | **6** |
| **Silverberg MJ. et al,2009** | * | * | * | * | * | * | * |  | **7** |
| **Serraino D et al, 2007** | * | * | * |  | * | * | * |  | **7** |
| **Hernandez-Ramirez RU. et al, 2017** | * | * | * | * | * |  | * |  | **5** |
| **Salters KA. et al, 2015** | * | * | * |  | * | * | * | * | **8** |
| **Godbole SV. et al, 2016** | * | * | * | * |  | * | * |  | **5** |
| **Piketty C et al, 2012** | * | * | * |  | * | * | * |  | **6** |
| **Seaberg EC. et al, 2010** | * | * | * | * | * | * | * |  | **6** |
| **Zhu W et al, 2019** | * | * | * |  | * | * | * |  | **7** |
| **Lee SO et al, 2022** | * | * | * | * | * | * |  |  | **5** |
| **Clark E et al, 2021** | * | * | * | * | * | * | * |  | **6** |
| **Park LS et al, 2016** | * | * | * |  | * |  | * |  | **6** |
| **Engsig FN. et al, 2011** | * | * | * |  | * |  | * |  | **5** |
| **Maso LD. et al, 2009** | * | * | * |  |  | * | * | * | **6** |
| **Chaturvedi AK et al, 2009** | * | * | * |  | * | * | * | * | **7** |
| **Engels EA et al, 2006** | * | * | * |  | * | * | * | * | **7** |
| **Tanaka LF et al, 2018** | * | * | * |  | * | * | * | * | **7** |

**
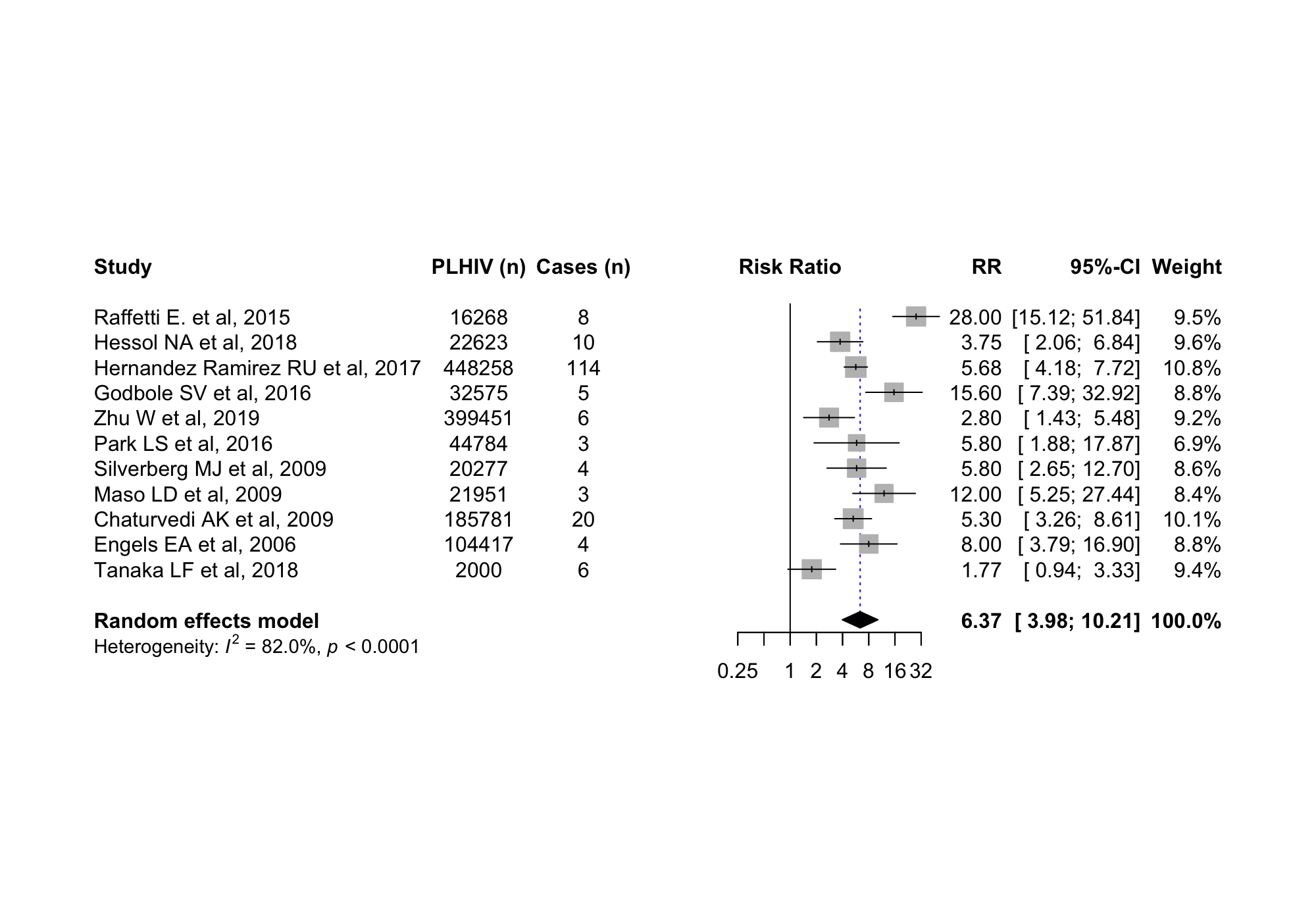

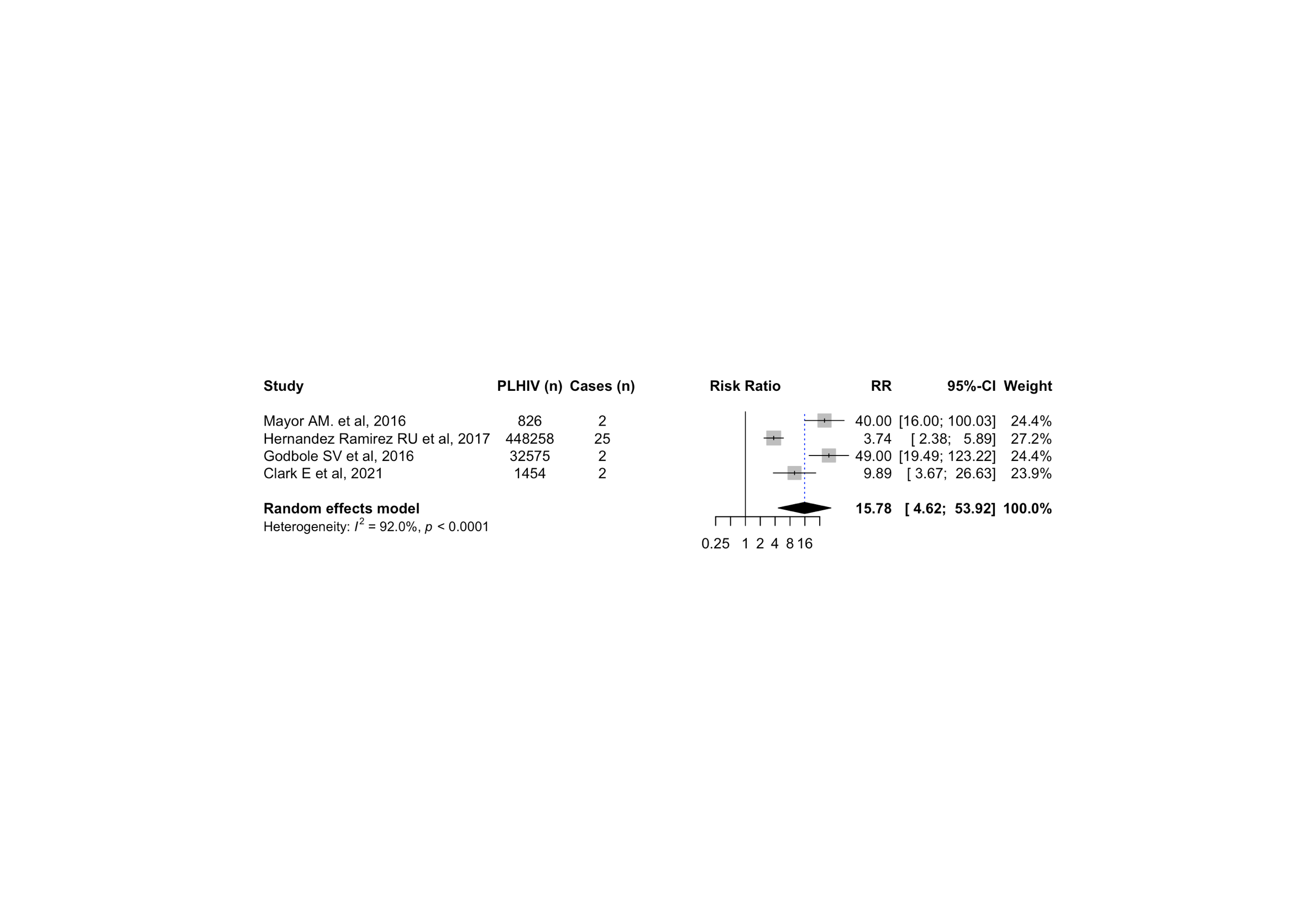

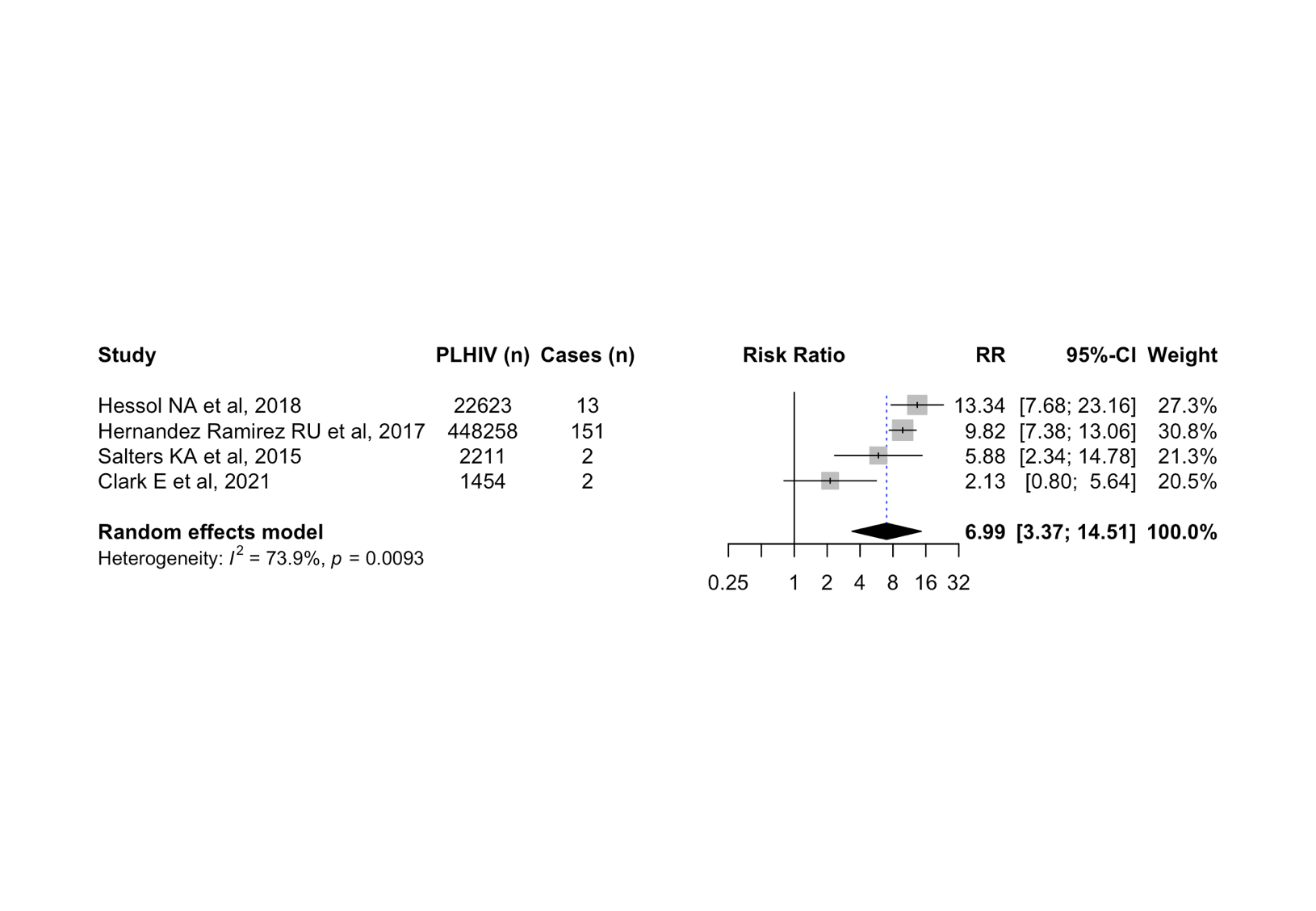
**

***Figure S2b:*** *Meta analysis of risk estimates and the confidence intervals weighted by inverse variance method for penile cancer*

***Figure S2a:*** *Meta analysis of risk estimates and the confidence intervals weighted by inverse variance method for vaginal cancer*

***Figure S2c****: Meta analysis of risk estimates and the confidence intervals weighted by inverse variance method for vulvar cancer*

**
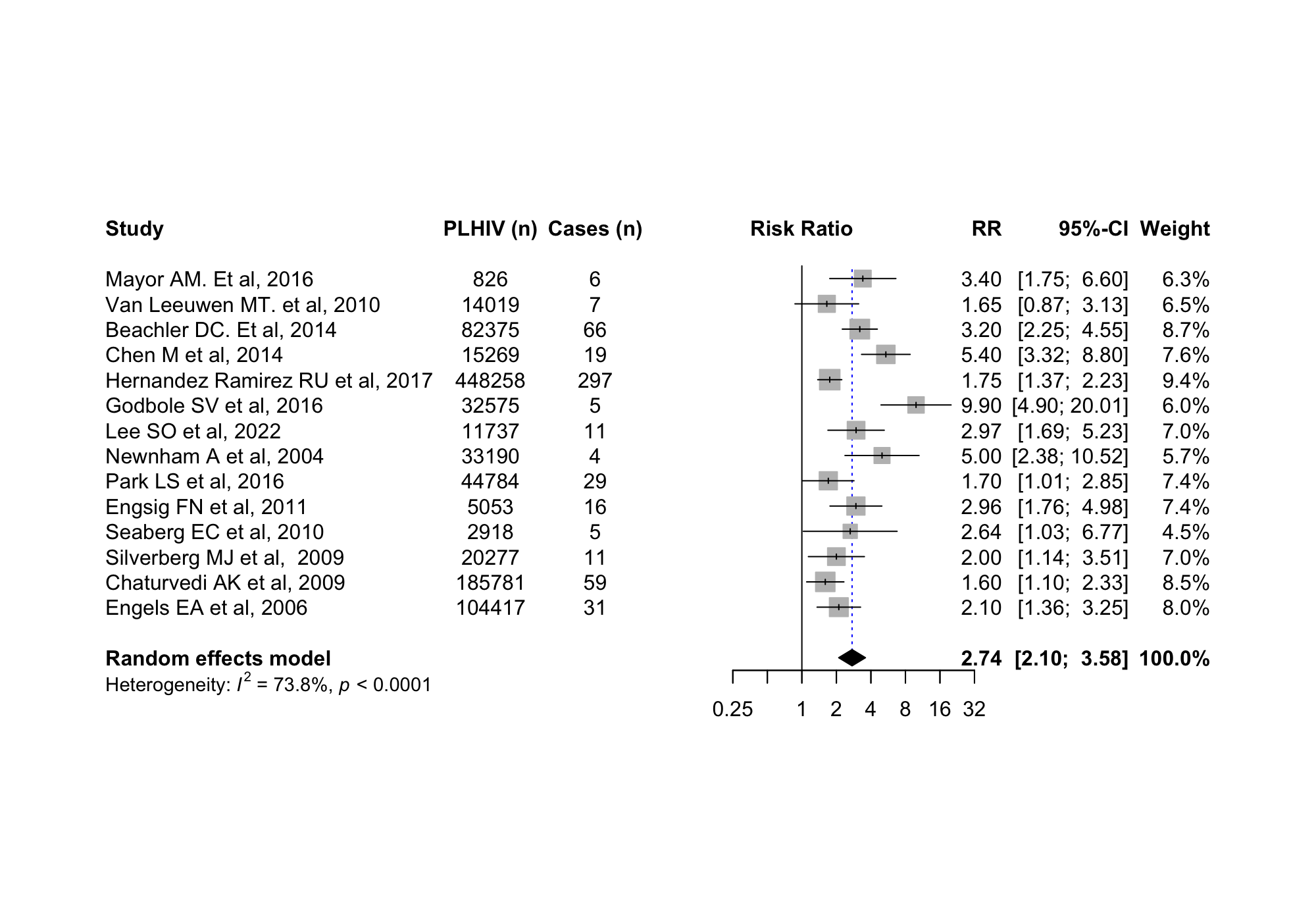
**

***Figure S2d:*** *Meta analysis of risk estimates and the confidence intervals weighted by inverse variance method for oropharyngeal cancer*

**
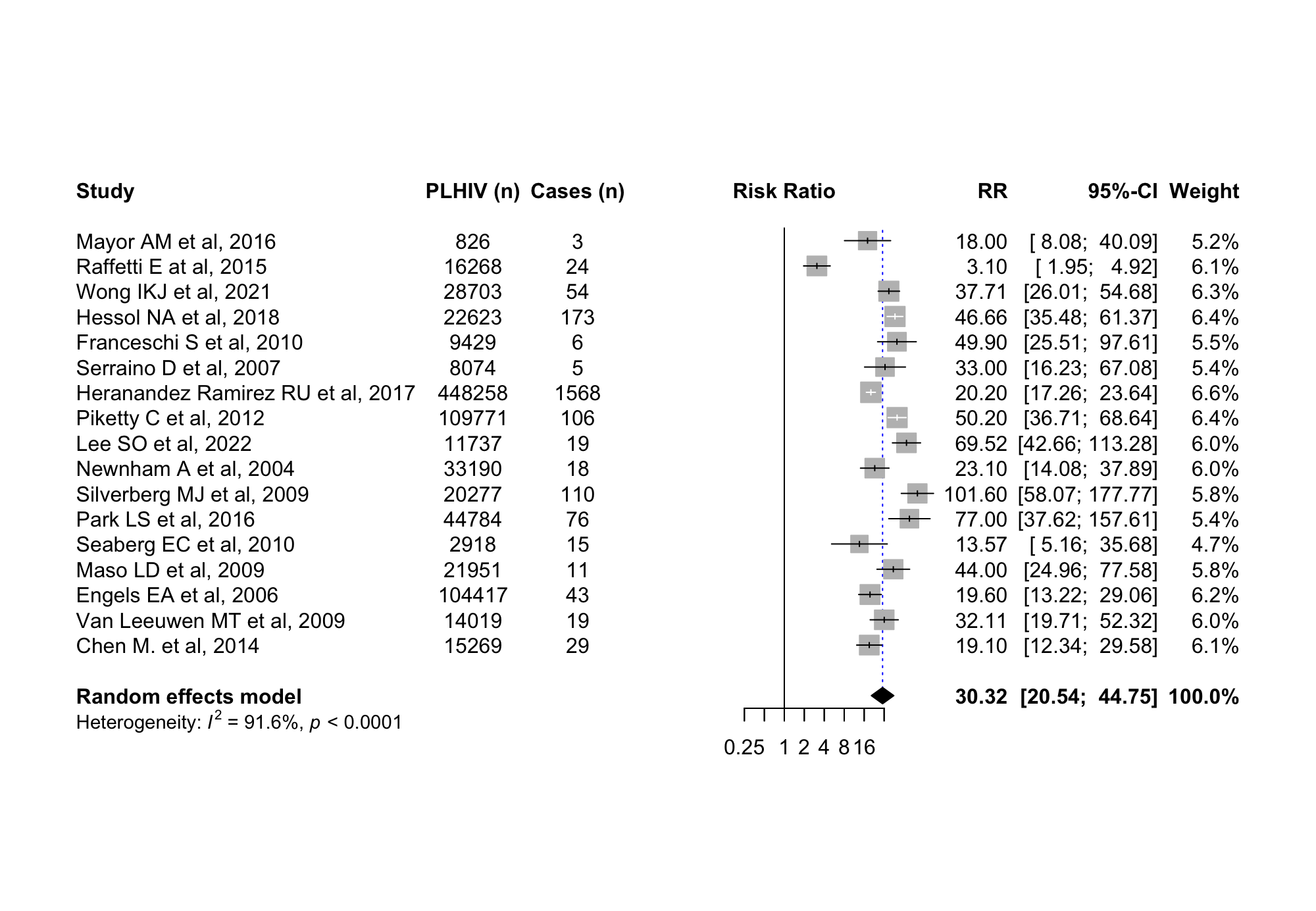
**

***Figure S2e****: Meta analysis of risk estimates and the confidence intervals weighted by inverse variance method for anal cancer*

**Table S6:** Sensitivity analyses removing studies high risk of bias and those pre-2014

| Cancer | Group | Number of studies | Pooled RR (95% CI) | *I^2^ (%)* | p-value |
| --- | --- | --- | --- | --- | --- |
| Oropharyngeal cancer | Pre 2014 | 7 | 3.62 | 87.2 | <0.01 |
|  | High risk of bias | 4 | 2.97 | 84.4 | <0.01 |
| Vulvar cancer | High risk of bias | 2 | 6.55 | 91 | <0.01 |
| Penile cancer | Pre 2014 | 4 | 6.93 | 88 | <0.01 |
|  | High risk of bias | 3 | 7.02 | 81.8 | <0.01 |
| Vaginal cancer | High risk of bias | 2 | 13.22 | 92.2 | <0.01 |
| Anal cancer | Pre 2014 | 9 | 30.11 | 91.7 | <0.01 |
|  | High risk of bias | 3 | 25.31 | 91.3 | <0.01 |

**
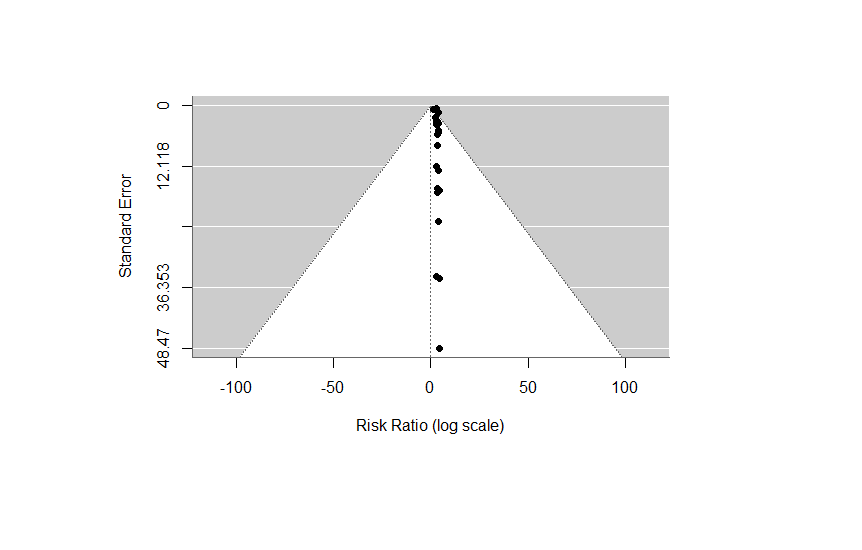
**

**Figure S3a:** Funnel plot assessing publication bias for papers reporting estimates on anal cancer. Log scale was used according to Meta-analysis R guide.

**
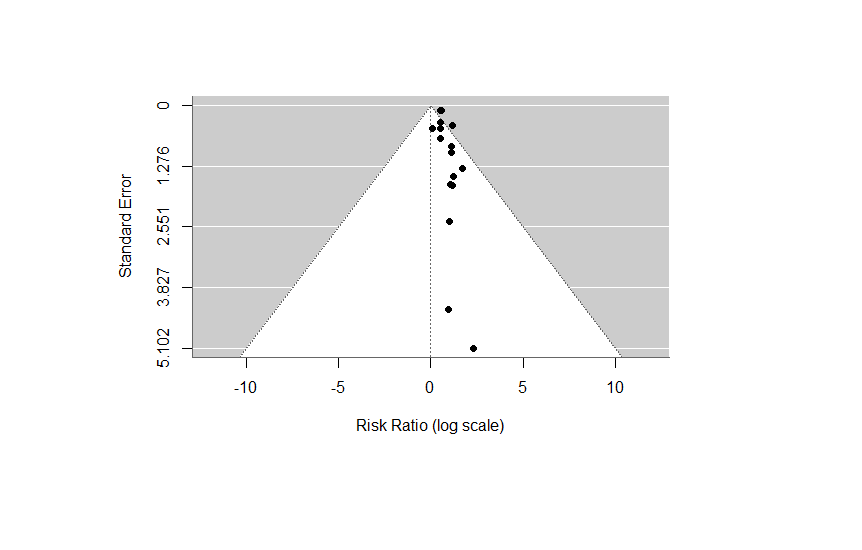
**

**Figure S3b:** Funnel plot assessing publication bias for papers reporting estimates on oropharyngeal cancer. Log scale was used according to Meta-analysis R guide.

***Figure S4a:*** *Graph showing the countries with the top 50 PAFs for anal cancer*

***Figure S4b:*** *Graph showing the countries with the top 50 PAFs for oropharyngeal cancer*

***Figure S4c:*** *Graph showing the countries with the top 50 PAFs for vaginal cancer*

***Figure S4d:*** *Graph showing the countries with the top 50 PAFs for vulvar cancer*

***Figure S4e:*** *Graph showing the countries with the top 50 PAFs for penile cancer*

**Table S7:** Countries with 0 vaccine coverage in the WHO’s HPV vaccine coverage dataset for 2022. These countries were assumed to have no national HPV vaccine programme, thus 0% vaccine coverage at the time of WHO data collection (2022), but some may have private and regional HPV programmes.

| **Countries with 0 vaccine coverage** | **Extra information** |
| --- | --- |
| Eswatini | Launched a HPV vaccine in schools programme in 2023 reaching 30,000 girls |
| Namibia | Aims to establish national HPV vaccine programme in 2025 |
| Equatorial Guinea | Conducted pilot HPV vaccine programmes in 2024 |
| Tanzania, United Republic of | 0% coverage, but introduced national vaccine programme in 2018 |
| Congo, Republic of |  |
| Congo, Democratic People Republic of | Aim to introduce in 2026 |
| Central African Republic |  |
| Gabon |  |
| Guinea-Bissau | Awaiting GAVI support |
| South Sudan |  |
| Togo | Introduced national programme in 2023 |
| Ghana |  |
| Guinea | Pilot scheme in 2024 |
| Chad |  |
| Burundi | Introduced national programme in 2024 |
| Mali |  |
| Sao Tome and Principe |  |
| Madagascar |  |
| Niger | Introduced national programme in 2024 |
| Comoros |  |
| Algeria |  |
| Angola |  |
| Benin |  |
| Nigeria | Introduced national programme in 2023 |
| Somalia |  |
| Morocco | Introduced national programme in 2022 |
| Haiti |  |
| Cuba |  |
| Nicaragua |  |
| Puerto Rico | Schools entry-requirement for HPV vaccines |
| Libya | Introduced in 2013, but no vaccine coverage data |
| Pakistan | Aim to introduce in 2025 |
| Kuwait |  |
| Qatar |  |
| Iraq |  |
| Jordan |  |
| Lebanon |  |
| Iran, Islamic Republic of |  |
| Tunisia | Aim to introduce in 2025 |
| Egypt | Privately available |
| Afghanistan |  |
| Sudan |  |
| Syrian Arab Republic | Planning to introduce with GAVI support |
| Yemen |  |
| Bahrain | Introduced in 2023 |
| Oman |  |
| Djibouti |  |
| Gaza Strip and West Bank |  |
| Russian Federation | Aim to introduce in 2026 with domestically produced quadrivalent |
| Belarus | Introduced in 2025 |
| Greece | Introduced in 2008, but no vaccine coverage data |
| Tajikistan |  |
| Croatia | Introduced in 2016, but no vaccine coverage data |
| Czechia |  |
| Azerbaijan |  |
| Montenegro | Introduced in 2022 |
| Romania | Introduced in 2023 |
| Poland | Introduced in 2023 |
| Bosnia Herzegovina | Regional vaccine programmes |
| Kazakhstan |  |
| Türkiye | Privately available |
| Ukraine | Aim to introduce in 2026 |
| Thailand | School programme introduced in 2017 |
| Timor-Leste | Introduced in 2024 |
| Bangladesh | Regional provisions with GAVI support |
| Nepal | Introduced in 2025 |
| India | Privately available and in regions |
| Papua New Guinea |  |
| Cambodia | Introduced in 2023 |
| Vietnam | Aim to introduce in 2026 |
| Philippines | Introduced in 2015, but no vaccine coverage data |
| China | Gradual roll out to certain regions |
| Mongolia |  |
| Korea, Republic of (South) | Introduced in 2016, but no vaccine coverage data |
| New Caledonia | School based programme |
| Vanuatu |  |
| French Polynesia | Introduced in 2023 |
| Korea, democratic People Republic of (North) |  |
| Guam | School based programme in 2016 for certain regions |

|  |
| --- |
|  |
|  |
|  |
|  |
|  |
|  |
|  |
|  |
|  |
|  |
|  |
|  |
|  |
|  |
|  |
|  |
|  |
|  |
